# FAM46C Is an Interferon-Stimulated Gene That Inhibits Lentiviral Particle Production by Modulating Autophagy

**DOI:** 10.1128/spectrum.05211-22

**Published:** 2023-06-26

**Authors:** Marilena Mancino, Giancarlo Lai, Federica De Grossi, Alessandro Cuomo, Lara Manganaro, Giacomo M. Butta, Ivan Ferrari, Elisa Vicenzi, Guido Poli, Elisa Pesce, Stefania Oliveto, Stefano Biffo, Nicola Manfrini

**Affiliations:** a INGM, Istituto Nazionale Genetica Molecolare Romeo ed Enrica Invernizzi, Milan, Italy; b Department of Biosciences, University of Milan, Milan, Italy; c Department of Experimental Oncology, IEO, European Institute of Oncology IRCCS, Milan, Italy; d Department of Pharmacological and Biomolecular Sciences, University of Milan, Milan, Italy; e Viral Pathogenesis and Biosafety Unit, San Raffaele Scientific Institute, Milan, Italy; f Vita-Salute San Raffaele University School of Medicine, Milan, Italy; Regional Centre for Biotechnology

**Keywords:** FAM46C, TENT5C, HIV-1, autophagy, interferons, lentiviruses, viral production

## Abstract

FAM46C is a multiple myeloma (MM) tumor suppressor whose function is only starting to be elucidated. We recently showed that in MM cells FAM46C triggers apoptosis by inhibiting autophagy and altering intracellular trafficking and protein secretion. To date, both a physiological characterization of FAM46C role and an assessment of FAM46C-induced phenotypes outside of MM are lacking. Preliminary reports suggested an involvement of FAM46C with regulation of viral replication, but this was never confirmed. Here, we show that FAM46C is an interferon-stimulated gene and that the expression of wild-type FAM46C in HEK-293T cells, but not of its most frequently found mutant variants, inhibits the production of both HIV-1-derived and HIV-1 lentiviruses. We demonstrate that this effect does not require transcriptional regulation and does not depend on inhibition of either global or virus-specific translation but rather mostly relies on FAM46C-induced deregulation of autophagy, a pathway that we show to be required for efficient lentiviral particle production. These studies not only provide new insights on the physiological role of the FAM46C protein but also could help in implementing more efficient antiviral strategies on one side and lentiviral particle production approaches on the other.

**IMPORTANCE** FAM46C role has been thoroughly investigated in MM, but studies characterizing its role outside of the tumoral environment are still lacking. Despite the success of antiretroviral therapy in suppressing HIV load to undetectable levels, there is currently no HIV cure, and treatment is lifelong. Indeed, HIV continues to be a major global public health issue. Here, we show that FAM46C expression in HEK-293T cells inhibits the production of both HIV and HIV-derived lentiviruses. We also demonstrate that such inhibitory effect relies, at least in part, on the well-established regulatory role that FAM46C exerts on autophagy. Deciphering the molecular mechanism underlying this regulation will not only facilitate the understanding of FAM46C physiological role but also give new insights on the interplay between HIV and the cellular environment.

## INTRODUCTION

FAM46C is an oncosuppressor gene which is found mutated in more than 10% of multiple myeloma (MM) patients (1–4). Both FAM46C mutations and loss of heterozygosity are associated with decreased overall survival of MM patients (5). We and others have demonstrated that expression of FAM46C triggers apoptosis of MM cells, while its depletion favors cell growth and survival (6, 7). FAM46C resides at the cytoplasmic side of the endoplasmic reticulum (ER) (6, 8), where it regulates intracellular vesicle trafficking, an event which in turn causes inhibition of autophagy. In the specific MM scenario, autophagic inhibition is deleterious for the cells, which ultimately undergo apoptosis (6).

FAM46C was suggested to function as an oncosuppressor not only in MM but also in colorectal (9), prostate (10), and gastric (11) cancer and in hepatocellular (12) and oral squamous cell (13) carcinoma. Moreover, FAM46C was recently proposed to function as a pan-cancer prognostic factor (14).

Outside of the tumoral environment, numerous evidence links FAM46C with regulation of viral replication and interferon (IFN) responses. FAM46C was, in fact, originally described by Schoggins et al. (15) as a type I IFN-stimulated gene (ISG). These authors, however, failed to confirm an antiviral activity for FAM46C but instead demonstrated that its expression enhanced the replication of yellow fever virus, West Nile virus, Venezuelan equine encephalitis virus, and chikungunya virus. FAM46C expression was shown to be upregulated in rice rats acutely infected by Andes virus (16), and, in a high-throughput image-based screening, FAM46C was found to inhibit the spread of influenza A virus in human lung adenocarcinoma cells (A549), an effect that was, however, not recapitulated in normal human bronchial epithelial cells (17). FAM46C was also shown to negatively affect the infectivity yield of Mason-Pfizer monkey virus and human endogenous retrovirus type K in MT4 human cells (18). In conclusion, FAM46C involvement in viral production is certain; what is less certain is FAM46C’s role upon viral infection, since its effects seem to be virus and/or cell specific.

Autophagy is a complex lysosomal degradation process through which cells maintain their homeostasis by eliminating/recycling intracellular components such as organelles or misfolded proteins. The selected material is targeted for autophagic degradation by a series of specific cargo receptors, among which the most characterized is p62 (19). The targeted material is then engulfed by a specialized double-membrane vesicle, the autophagosome, which is composed by several accessory proteins, including lipidated LC3 (LC3-II). After maturation, autophagosomes fuse with lysosomes, and their contents are then degraded and recycled.

It has long been known that autophagy cross talks with the immune system in order to regulate antiviral responses as it (i) triggers the innate immune response in order to induce IFN production (20), (ii) favors antigen processing and presentation (21), and (iii) targets intruding viruses by direct degradation of viral components. For these reasons, viruses have evolved mechanisms to evade/inhibit autophagic targeting or even to hijack autophagosomes in order to favor their own replication (22). In this scenario, the autophagic pathway has been widely described as involved in both early and late events of human immunodeficiency virus (HIV) infection (22) but was shown to be able to differentially inhibit or favor viral replication, depending on the cell type analyzed (23).

Here, we provide evidence that in HEK-293T cells autophagy has a proviral function. In this context FAM46C functions as an inhibitor of HIV spread. In particular, we show that, in human HEK-293T cells, wild-type FAM46C (wt-FAM46C), but none of the most frequently found mutant variants of the protein, inhibits HIV-derived and HIV lentiviral particle production. This effect requires the ability of FAM46C to inhibit autophagy, since autophagic stimulation totally abolishes this FAM46C-induced phenotype, and possibly relies on the established role of FAM46C in regulating intracellular trafficking/dynamics, as we show that the interaction of FAM46C with several cytoskeleton and intracellular vesicle components is altered during viral production. Finally, we link regulation of FAM46C expression with autophagic inhibition by formally demonstrating that FAM46C is both a type I and a type II ISG.

## RESULTS

### FAM46C inhibits lentiviral production in *cis*.

**FIG 1:**
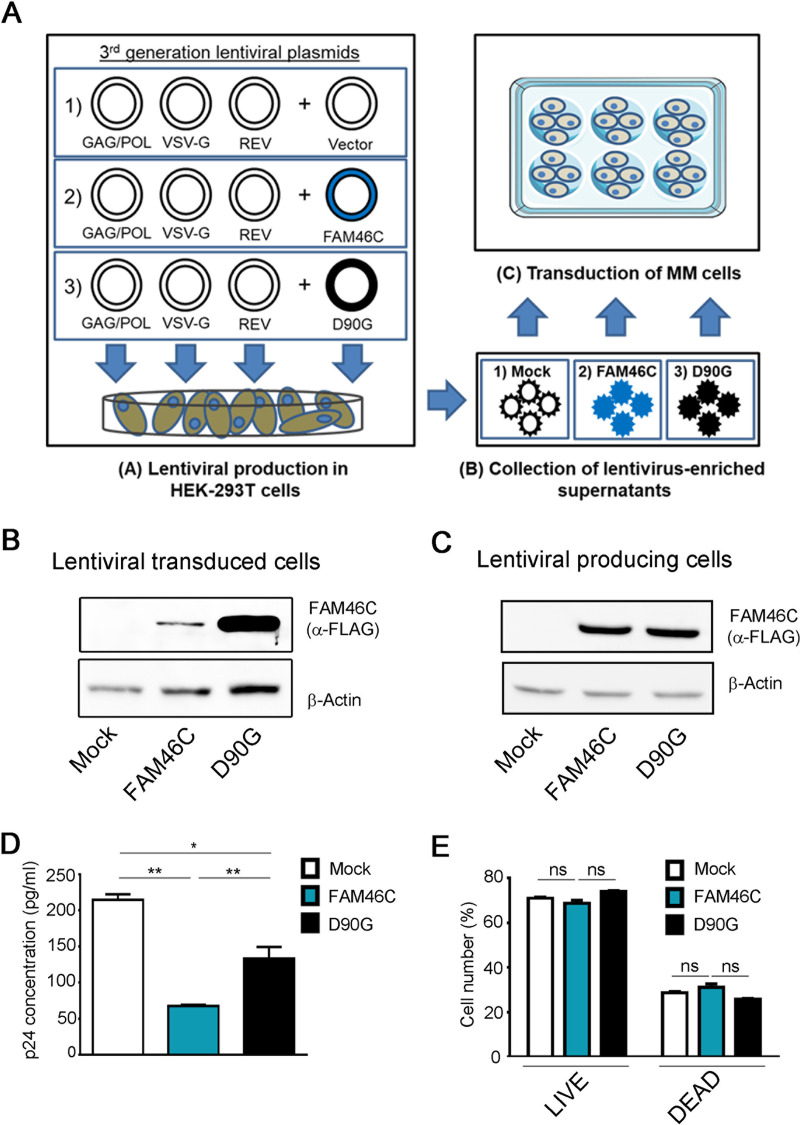
FAM46C inhibits lentiviral particle production in *cis*. (A) Cartoon depicting our viral particle production and transduction approach. (Subpanel A) HEK-293T cells were transfected with plasmids encoding for the lentiviral GAG/POL, VSV-G, and REV genes, together with plasmids encoding for either the FAM46C gene, the D90G mutant allele, or an empty vector. (Subpanels B and C) The viral particles produced were then collected from the supernatant (subpanel B) and used to infect OPM2 MM cells (subpanel C). (B) Western blot analysis showing FAM46C and D90G protein levels in OPM2 MM cells. OPM2 cells were transduced with FAM46C-, D90G-, or empty vector-expressing lentiviruses as described in panel A. Cytoplasmic lysates derived from the samples were subjected to SDS-PAGE and Western blotting with α-FLAG antibodies. β-Actin was used as a loading control. (C) Western blot analysis showing FAM46C and D90G protein levels in virus-producing HEK-293T cells. HEK-293T cells were transfected to produce lentiviral vectors expressing either FAM46C, the D90G mutant allele, or an empty vector, as described in panel A. Samples were then prepared as described in panel B. β-Actin was used as a loading control. (D) p24 concentrations in the lentivirus-enriched supernatants produced by HEK-293T cells. The lentivirus-containing supernatants produced by the cells described in panel C were analyzed for p24 levels by ELISA. (E) Percentage of live and dead HEK-293T cells during lentiviral production. The cells described in panel C were used to assess cell viability through flow cytometry. Blots are representative of three independent experiments. Histograms represent the means ± the SD of three independent experiments. Statistical *P* values were calculated using double-tailed unpaired *t* tests. ns, *P > *0.05; **, *P < *0.01.

Recently, we have demonstrated that FAM46C is a novel oncosuppressor which regulates intracellular trafficking, secretion, and autophagy in MM cells (6). During our characterization of FAM46C function, we used HIV-1-based lentiviral vectors to reconstitute, in MM cells lacking physiological expression of FAM46C, wt-FAM46C (here referred to as FAM46C) or, as controls, either an empty vector or the loss-of-function D90G-FAM46C mutant allele, which harbors one of the amino acid (aa) substitutions most frequently found in MM patients (here referred to as D90G) (1, 6) (Fig. 1A). We serendipitously found that, despite being transduced with same volumes of supernatant-containing lentiviral particles, MM cells transduced with FAM46C-lentiviral vectors expressed much lower protein levels compared to cells transduced with D90G-lentiviral vectors (Fig. 1B). To test whether this effect was due to a defective production of FAM46C-expressing viral particles from HEK-293T cells, rather than the production of nonfunctional lentiviral particles, after transfecting cells with either the p-lenti wt-FAM46C, the p-lenti-D90G or an empty vector along with lentiviral envelope and packaging plasmids, we measured the efficiency of viral particle production by quantifying p24 viral capsid abundance in the cell-free supernatant.

Although FAM46C and D90G were expressed similarly in HEK-293T cells during lentiviral production (Fig. 1C), we found that the quantity of wt-FAM46C-expressing lentiviruses detected in the supernatant was considerably lower compared to that of D90G-expressing lentiviruses (Fig. 1D), suggesting that expression of wt-FAM46C caused inefficient viral particle production. Intriguingly, the production of lentiviruses expressing an empty vector was more efficient than that of either FAM46C- or D90G-expressing lentiviruses, possibly due to residual activity of the D90G mutant allele.

In order to exclude the possibility that the reduced viral titer of FAM46C-expressing viruses could be the consequence of cytotoxicity, we monitored the viability of lentivirus-producing cells during virus production. We detected no difference in the percentage of dead cells between HEK-293T cells expressing FAM46C, the D90G mutant, or an empty vector (Fig. 1E), indicating that FAM46C expression is *per se* not cytotoxic to HEK-293T cells, in accordance with previously published data (6) and confirming that the reduced titer of FAM46C-expressing lentiviruses was due to a defect in lentiviral particle production.

To investigate whether abrogation of FAM46C antiviral activity was an effect due to the specific D90G aa substitution or a more general feature related to FAM46C loss of function, we checked the effect of other mutant alleles of FAM46C known to abolish FAM46C-induced phenotypes. Among the many other substitutions found in MM patients, we selected the F184L and Y291C substitutions due to their relatively high occurrence (1, 4). After concomitant transfection of HEK-293T cells with envelope and packaging vectors and constructs to express the FAM46C-F184L and FAM46C-Y291C variants, we measured the efficiency of lentiviral particle production as previously described. Despite comparable levels of expression of wt and mutant FAM46C proteins in lentivirus-producing cells (see Fig. S1A in the supplemental material), the viral titer of the lentiviruses expressing the mutant protein versions of FAM46C was much higher compared to that of viruses expressing wt-FAM46C, indicating that both aa substitutions abrogated FAM46C capability to efficiently inhibit lentiviral production (see Fig. S1B). These results suggest that FAM46C has an antiviral activity and that the most common FAM46C loss-of-function mutations, at least in part, abrogate it.

### FAM46C inhibits lentiviral production in *trans*.

**FIG 2:**
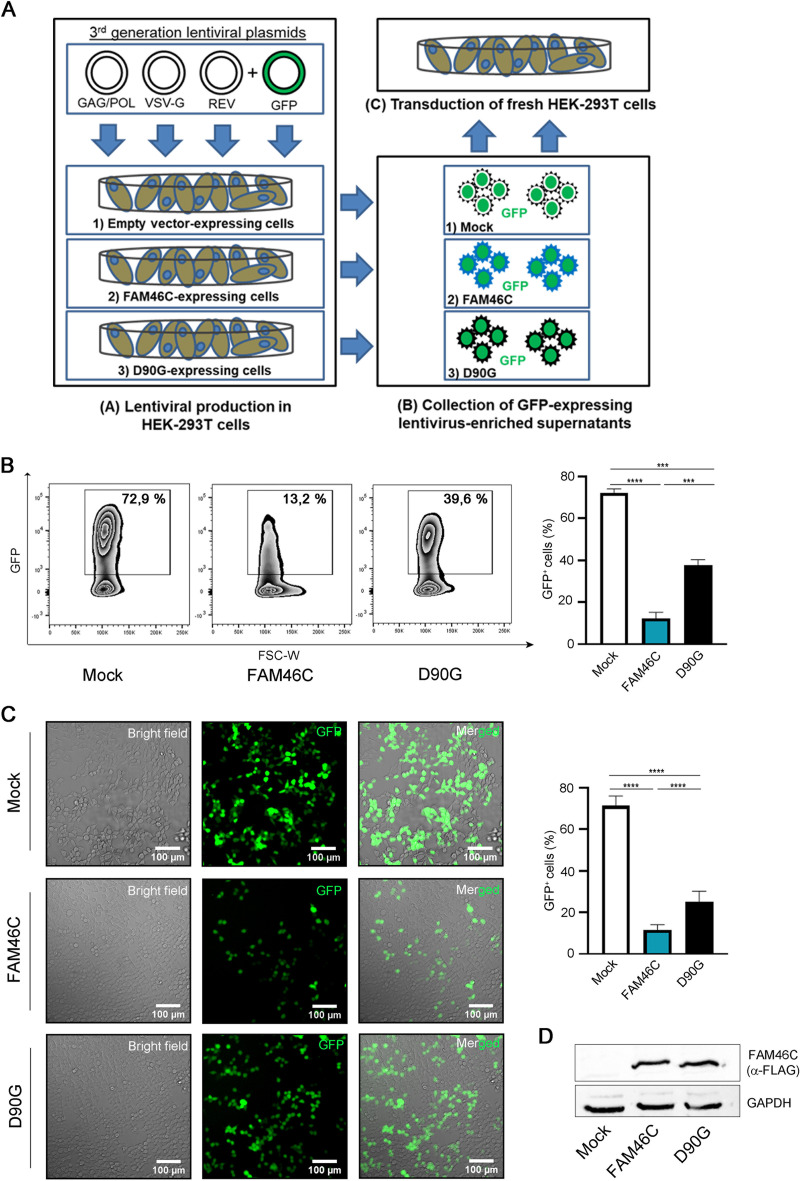
FAM46C inhibits lentiviral particle production in *trans*. (A) Cartoon depicting our viral particle production and transduction approach. (Subpanel A) HEK-293T cells expressing either an empty vector, FAM46C, or the D90G mutant were transfected with plasmids encoding for the lentiviral GAG/POL, VSV-G, and REV genes and GFP. (Subpanels B and C) The GFP-expressing lentiviral particles produced were then collected from the supernatant (subpanel B) and used to infect fresh HEK-293T cells (subpanel C). (B) Quantitation of GFP^+^ HEK-293T cells after transduction with GFP-expressing lentiviruses. GFP-expressing lentiviruses produced in FAM46C-, D90G-, or empty vector-expressing HEK-293T cells were used to infect fresh HEK-293T cells as described in panel A. The number of GFP^+^ cells was then assessed by flow cytometry. (Left) Representative zebra plots showing the percentage of GFP^+^ cells. (Right) Histograms representing the means ± the SD of three independent experiments. (C) Fluorescence microscopy analysis of HEK-293T cells transduced with GFP-expressing lentiviruses. The cells in panel B were analyzed by fluorescence microscopy. (Left) Representative confocal microscopy images. (Right) Histograms representing the mean percentage of GFP^+^ cells ± the SD of 12 independent fields of view. (D) Western blot analysis showing FAM46C and D90G protein levels in virus-producing HEK-293T cells. HEK-293T cells were transfected to produce GFP-expressing lentiviral particles in the presence of either FAM46C, the D90G mutant, or an empty vector, as described in panel A. Cytoplasmic lysates derived from the samples were subjected to SDS-PAGE and Western blotting with α-FLAG-antibodies. GAPDH was used as a loading control. Statistical *P* values were calculated using double-tailed unpaired *t* tests. ***, *P < *0.001; ****, *P < *0.0001.

To rule out the possibility that the low yield of wt-FAM46C virus production was due to *cis*-acting vector-intrinsic factors, we expressed the FAM46C protein in *trans*. During production of green fluorescent protein (GFP)-expressing lentiviruses, HEK-293T cells were transiently cotransfected with nonlentiviral plasmids expressing either FAM46C, D90G, or an empty vector (Fig. 2A). GFP-lentivirus-containing supernatants were collected 48 h posttransfection and then used in equal volumes to reinfect fresh HEK-293T cells. Viral production was determined by quantifying the number of GFP^+^ HEK-293T target cells by flow cytometry 5 days postransduction. As shown in Fig. 2B and C, a significantly lower percentage of GFP-positive cells was obtained after transduction with lentiviral particles produced in HEK-293T cells expressing FAM46C compared to that obtained after transduction with lentiviruses produced in cells expressing either the D90G mutant or the empty vector control. This result was not dependent on differential transfection efficiency of virus-producing cells, since FAM46C and D90G protein levels were comparable in FAM46C- and D90G-expressing cells (Fig. 2D). Similar results were also obtained when we repeated the experiment in genetically modified HEK-293T cell lines harboring an inducible promoter to regulate the expression of FAM46C or of the D90G mutant (see Fig. S2A to D), unequivocally demonstrating that FAM46C is able to inhibit HIV-1-based lentiviral vector production also in *trans* and ruling out any bias due to vector-intrinsic factors.

### FAM46C inhibits viral production by limiting viral gene expression at the posttranslational level.

**FIG 3:**
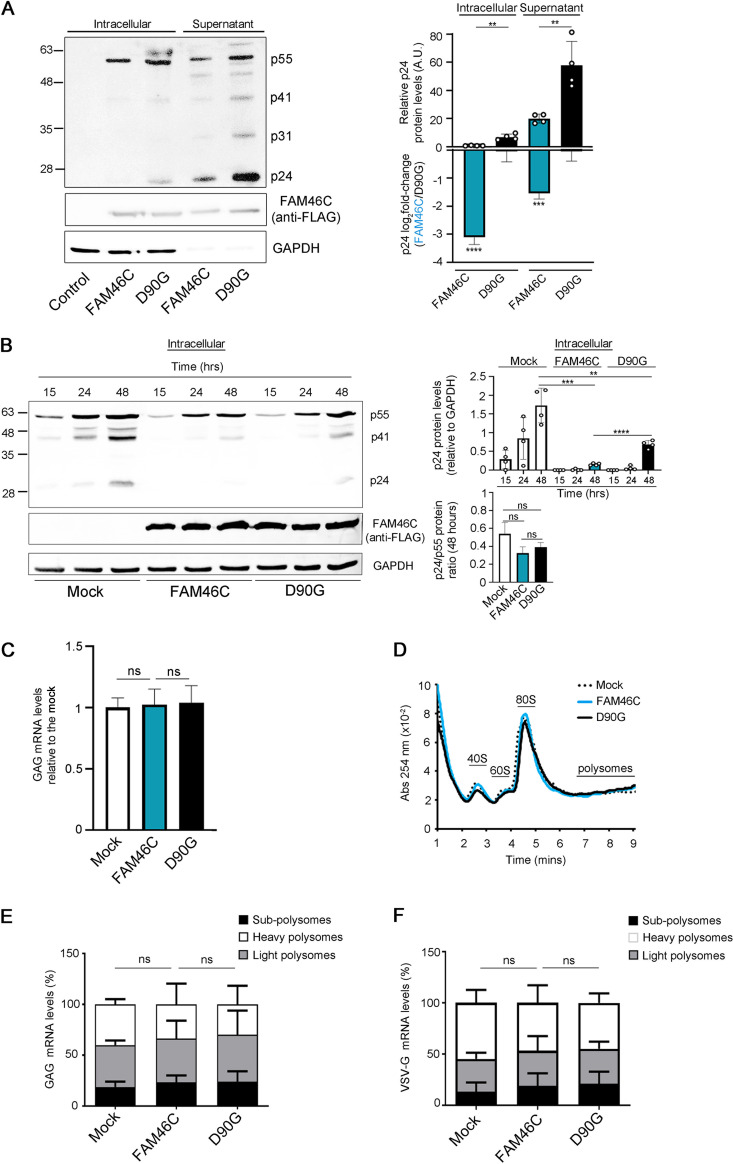
FAM46C inhibits lentiviral particle production by regulating viral gene expression at the posttranslational level. (A, left) Representative western blot showing the levels of viral proteins both in the intracellular compartment and in the supernatant of viral-producing HEK-293T cells. HEK-293T cells were transfected as described in Fig. 1A. Protein samples were extracted and subjected to SDS-PAGE and western blotting with the antiserum of an HIV-1-infected patient. GAPDH was used as a loading control. (Right) Histograms representing absolute and relative p24 levels. (B, left) Representative western blot showing the levels of intracellular GAG proteins in virus-producing HEK-293T cells. HEK-293T cells were transfected as described in Fig. 2A, and GAG protein levels were detected by western blotting following SDS-PAGE on protein lysates at the indicated time points after transfection using α-p24 antibodies. GAPDH was used as a loading control. (Right) Histograms representing relative p24 levels and p24/p55 ratios. (C) GAG mRNA levels in HEK-293T lentivirus-producing cells. HEK-293T cells were transfected as described in Fig. 2A, and GAG mRNA levels were determined by RT-qPCR. (D) Polysome profiles of HEK-293T lentivirus-producing cells. The cells in panel C were lysed, and polysome profiles were isolated and analyzed. (E and F) Levels of GAG (E) and VSV-G (F) viral transcripts in different polysome fractions of lentivirus-producing HEK-293T cells. Polysome fractions from the samples described in panel D were purified and assessed for transcript abundance. Histograms represent the means ± the SD of three independent experiments. Blots are representative of three independent experiments. Statistical *P* values were calculated using double-tailed unpaired *t* tests. ns, *P > *0.05.

In MM, FAM46C alters protein stability and secretion by affecting intracellular trafficking (6). To determine whether wt-FAM46C was (i) affecting viral protein production or (ii) simply inhibiting viral particle release, we measured the amount of both intracellular and extracellular viral proteins during lentiviral production in HEK-293T cells. Antibody-containing serum from an HIV-infected patient was used to detect viral proteins both in the intracellular compartment and in the cell-free supernatant of *in vitro* cultured lentivirus-producing cells. As expected, we found a strong reduction of viral proteins in the cell-free supernatant of wt-FAM46C virus-producing cells compared to cells producing FAM46C-D90G lentiviruses (Fig. 3A). Such proteins included the GAG precursor p55, its processing derivatives p41 and capsid protein p24, and viral integrase p31. We detected a reproducible difference also when we analyzed the amount of intracellular viral proteins (Fig. 3A), suggesting that wt-FAM46C acts by inhibiting overall production of lentiviral particles rather than affecting only virus release from the cell. p24 is the capsid protein required for viral particle production and derives from the processing of the Gag p55 precursor protein (24). To exclude the possibility that the reduced intracellular levels of p24 that we detected were the consequence of defective p55 processing, we specifically monitored the accumulation of intracellular p55 and p24 over time during GFP lentivirus production in HEK-293T cells expressing either FAM46C, the D90G mutant, or an empty vector (Fig. 3B). As expected, FAM46C-expressing lentivirus-producing cells accumulated over time much less p24 protein compared to either D90G- or empty vector-expressing cells (Fig. 3B), but such reduced levels were always coupled by overall reduced p55 levels, suggesting no significant defect in p55 processing in FAM46C-expressing cells. This result was further confirmed when we quantified the p55/p24 protein ratio at each time point analyzed and found no difference between FAM46C-, D90G-, and empty vector-expressing lentivirus-producing cells (Fig. 3B, right). Notably, although D90G-expressing cells always produced more viral proteins than did FAM46C-expressing cells, they also always produced less viral proteins compared to empty vector-expressing cells (Fig. 3B), confirming the idea that, in this scenario, the D90G substitution generates only a partial loss-of-function protein.

Next, to be certain that the effects we were seeing were not specific of laboratory-engineered derivatives of HIV-1 but could hold true also for wt HIV-1, we checked whether and how FAM46C expression could affect the production of different HIV-1 clones. We produced viral particles in HEK-293T cells coexpressing either FAM46C, the D90G mutant allele, or an empty vector and monitored intracellular and extracellular viral protein levels (see Fig. S3A to C). We found that, in all of the clones tested, FAM46C had an inhibitory effect on viral particle production, as determined by analyzing the levels of viral proteins in the viral particle-containing supernatant, confirming that FAM46C is capable to inhibit also HIV-1 replication (see Fig. S3A to C).

The inhibitory effect of FAM46C was seen also intracellularly, corroborating the fact that FAM46C affects viral protein production rather than viral particle release from the cell. When we analyzed p55 and p24 levels, we confirmed that FAM46C had no effect in p55 processing as the p24/p55 ratios were overall comparable between FAM46C-expressing, D90G-expressing, and control cells (see Fig. S3B, right, and Fig. S3C, bottom). Taken together, these results confirmed that the inhibitory effect of FAM46C on virus production is not exerted through regulation of Gag p55 precursor protein processing but rather on general viral protein production and that this effect is broad and involves also HIV-1. To further validate and strengthen the idea of a broad antiviral function of FAM46C, we tested the effect of FAM46C downmodulation on viral replication in a CD4^+^ T cell line (SUP-T1) infected with HIV-1 NL4-3 nef-IRES GFP (25). We found that FAM46C downmodulation slightly, but significantly, increased viral replication capacity compared to a cell line harboring physiological FAM46C levels (see Fig. S3D), suggesting that the presence of wt-FAM46C is indeed responsible for inhibiting replication also of replication-competent HIV-1 particles.

Next, we investigated how FAM46C was regulating intracellular viral protein levels. Since FAM46C was recently proposed to function as a noncanonical poly(A) polymerase affecting specific transcript stability in certain environments (26), we first checked whether the reduction of intracellular viral proteins could be the direct consequence of reduced viral mRNA abundance. Reverse transcription-quantitative PCR (RT-qPCR) showed that lentivirus-producing cells expressing either wt-FAM46C, the D90G mutant variant, or an empty vector had similar levels of intracellular GAG mRNA (Fig. 3C), indicating no effect on GAG mRNA abundance.

Next, we analyzed whether FAM46C expression could regulate overall translational rates during lentiviral particle production. We performed polysome profiling and puromycin incorporation experiments in HEK-293T cells transfected with an empty vector, wt-FAM46C or the D90G mutant allele in the presence of lentiviral envelope and packaging vectors. Polysome profiles (Fig. 3D) and puromycin incorporation efficiencies (see Fig. S4A) were similar under all conditions analyzed, indicating no overall difference in global mRNA translation, in accordance with previous results (6). Moreover, when we checked for polysome-enriched viral mRNA levels, we found no difference between FAM46C-, D90G-, or empty vector-expressing cells (Fig. 3E and F), indicating that reduced intracellular lentiviral protein levels did not depend on defective translation of viral transcripts. Altogether, these results suggest that FAM46C inhibits viral replication by regulating viral gene expression at the posttranslational level.

### FAM46C affects viral production by negatively regulating autophagy.

**FIG 4:**
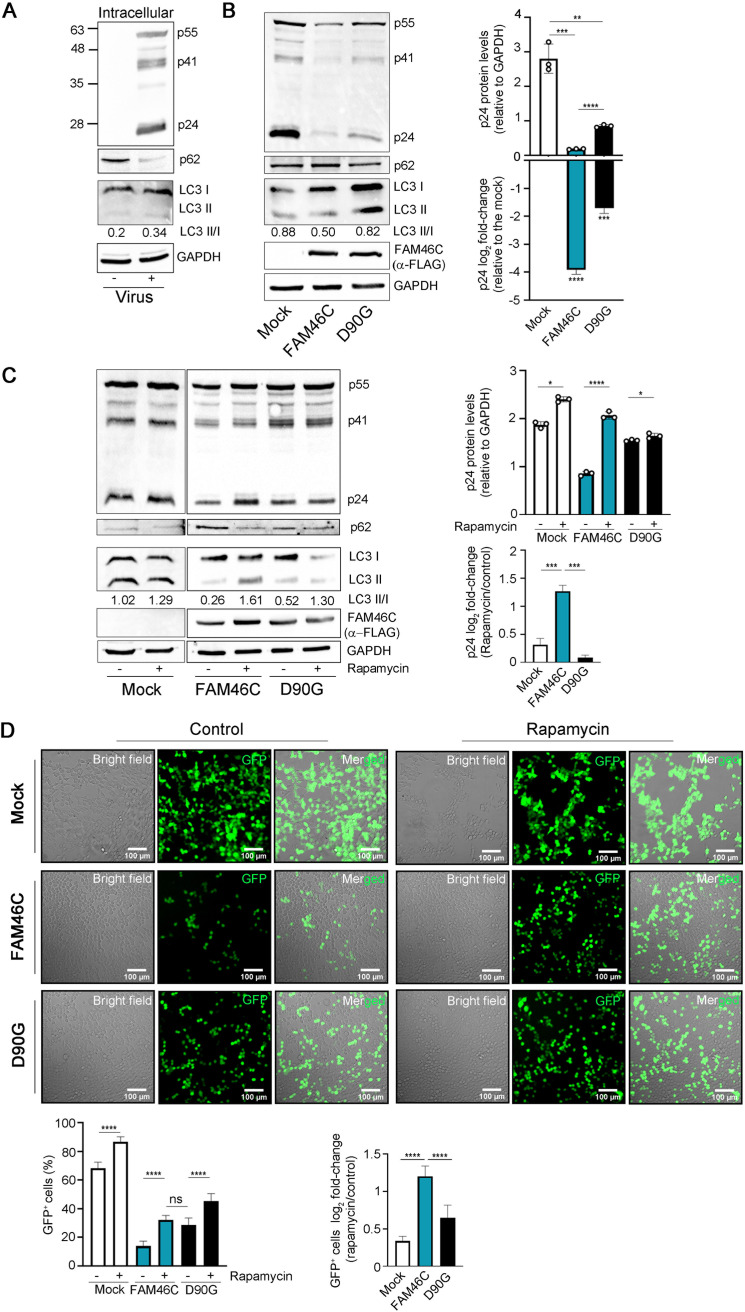
Autophagy is required for FAM46C-induced inhibition of viral particle production. (A) Representative western blot showing the levels autophagic markers and Gag proteins in virus-producing HEK-293T cells. HEK-293T cells were transfected with lentiviral envelope and packaging plasmids only. Cytoplasmic lysates derived from the samples were subjected to SDS-PAGE and western blotting with α-p24 and α-p62 antibodies. GAPDH was used as loading control. (B, left) Representative western blot showing the levels autophagic markers and Gag proteins in virus-producing HEK-293T cells. HEK-293T cells were transfected as described in Fig. 2B. Cytoplasmic lysates derived from the samples were subjected to SDS-PAGE and western blotting with α-p62 and α-LC3-II α-p24 and α-FLAG antibodies. GAPDH was used as a loading control. (Right) Histograms representing absolute and relative p24 levels. (C, left) Representative western blot showing the levels autophagic markers and Gag proteins in HEK-293T cells transfected as in panel A but either treated or not treated 24 h posttransfection with 100 nM rapamycin for an additional 24 h. Samples were prepared as in panel A. (Right) Histograms representing absolute and relative p24 levels. (D) Fluorescence microscopy analysis of HEK-293T cells transduced with GFP-expressing lentiviruses. GFP-expressing lentiviruses produced in FAM46C-, D90G-, or empty vector-expressing HEK-293T cells either treated or not treated with rapamycin were used to infect fresh HEK-293T cells. (Top) Representative confocal microscopy images. (Bottom) Histograms representing either the mean percentage or the log_2_-fold change of GFP^+^ cells ± the SD of 14 independent fields of view. Statistical *P* values were calculated using double-tailed unpaired *t* tests. ns, *P* > 0.05; ***, *P < *0.001; ****, *P < *0.0001.

Recently, we have demonstrated that FAM46C expression indirectly inhibits autophagy (6), a pathway often involved in regulating viral replication. Hence, we hypothesized that autophagic deregulation might have been involved in FAM46C-induced reduction of lentiviral protein intracellular accumulation. Autophagy can affect viral replication in several ways: in some cases, it functions as a protection mechanism for the host cell, impeding virion formation/egress, while for some viruses, it is required for proper replication/cell release (22). At first, we defined whether autophagy was altered during routine lentiviral production in HEK-293T cells by monitoring the levels of p62, an autophagosome cargo protein that is degraded by autophagy. We found that p62 levels drastically diminished in lentivirus-producing HEK-293T cells compared to control cells (Fig. 4A), suggesting autophagic augmentation. Accordingly, when we monitored the level of lipidated LC3, a marker of autophagosome formation, by quantifying the ratio of the lipidated versus nonlipidated form of the protein (LC3B-II/LC3-I), we found that it actually increased, suggesting that autophagy is indeed activated/required during lentiviral production in HEK-293T cells. Next, we monitored whether there was an alteration of the autophagic state during lentiviral production in FAM46C-expressing compared to D90G-expressing or control cells. In concomitance with a reduction of intracellular p24, cells expressing FAM46C actually accumulated more p62 compared to control cells or cells expressing the D90G allele (Fig. 4B), pinpointing autophagic inhibition. Accordingly, when we monitored the level of lipidated LC3, we found that it was reduced in FAM46C-expressing compared to D90G-expressing or control cells (0.5 versus 0.82 or 0.88, respectively), indicating that, during lentiviral production, autophagy is actually reduced in cells that express FAM46C.

Next, we tested whether this defect in autophagy could account for the inefficient viral particle production caused by FAM46C expression. We induced autophagy, through mTORC1 inhibition using rapamycin (27), during GFP lentiviral particle production in HEK-293T cells expressing either FAM46C, the D90G mutant, or an empty vector and monitored (i) intracellular viral protein production through p24 detection and (ii) overall viral particle production by cell transduction using the virus-enriched supernatant produced by the cells, as previously described (Fig. 2A). In all the virus-producing cell types, rapamycin treatment reduced the intracellular levels of p62 and increased the LC3-II/LC3-I ratio, confirming rapamycin-dependent induction of autophagy (Fig. 4C). The extent of such effect was, however, gene dependent and inversely proportional with the autophagic levels of each cell type. Autophagy was indeed induced most efficiently in FAM46C-expressing cells (>5-fold LC3-II/LC3-I increase), where autophagy is the least active; less efficiently in D90G-expressing cells (3-fold LC3-II/LC3-I increase), where autophagy is only partially dampened compared to the control; and least efficiently in empty vector-expressing cells (only 30% LC3II/I increase), where autophagy is most active. Rapamycin partially suppressed the virus production defect induced by FAM46C, since it drastically increased both the intracellular levels of p24 (Fig. 4C) and the quantity of viral particles produced by FAM46C-expressing cells, making them similar to those of D90G-expressing cells (Fig. 4D). Although an increase in viral production was also detectable in cells expressing either the D90G allele or an empty vector (Fig. 4C and D; see also Fig. S4B and C in the supplemental material), such an increase was milder, in line with their less responsiveness to rapamycin-dependent autophagic stimulation (Fig. 4C).

To confirm that FAM46C was inhibiting viral production via autophagic regulation, we produced stable HEK-293T cell lines downmodulated for upstream autophagy regulator ATG5 (see Fig. S4D) and tested the viral production capability of cells expressing either FAM46C, the D90G mutant allele, or a control vector (see Fig. S4E). As expected, ATG5 depletion drastically inhibited autophagy, since its downmodulation caused an increase of p62 and a decrease of LC3-II (see Fig. S4D). Autophagic inhibition was coupled by a reduction in viral protein production in all the cell lines analyzed (see Fig. S4E), confirming the requirement of autophagy for proper lentiviral protein production in HEK-293T cells. However, ATG5 downmodulation inhibited viral protein production more efficiently in control and D90G-expressing cells compared to FAM46C cells, indicating that FAM46C expression renders cells less responsive to autophagic inhibition, a result which is in agreement with the low viral protein production capacity and defective autophagic activation of FAM46C cells. Given the tight cross talk between autophagic and proteasomal degradation, that autophagic inhibition is known to upregulate proteasomal activation (28), and that the ubiquitin-proteasome system is a known regulator of viral entry and replication (29, 30), we checked whether proteasome-dependent protein degradation could explain the reduced quantity of viral proteins found in FAM46C-expressing cells. We treated lentivirus-producing HEK-293T cells expressing either FAM46C, the D90G mutant, or an empty vector, with proteasome inhibitor bortezomib (BTZ) and assessed the accumulation of intracellular viral proteins. BTZ treatment caused an increase in the quantity of viral proteins in all of the cell lines analyzed (see Fig. S4F), suggesting that, in this scenario, viral protein turnover is, at least in part, dependent on proteasomal regulation. Intriguingly, the effect was more pronounced in FAM46C-expressing cells, indicating that, when autophagy is reduced, viral protein degradation is more reliant on the proteasome. In line with this evidence, BTZ treatment was capable to partially suppress FAM46C-induced inhibition of viral protein production (see Fig. S4F).

Overall, these results suggest that the reduced levels of viral proteins found in FAM46C-expressing cells are, at least in part, dependent on proteasomal degradation. In summary, we found that, during lentiviral production in HEK-293T cells, autophagy has a proviral function, that expression of FAM46C inhibits viral replication by inhibiting autophagy, and that proteasomal degradation of viral proteins is involved in this mechanism.

### The interactome of FAM46C drastically varies upon lentiviral production and comprises components of intracellular vesicles and proteins involved in intracellular trafficking.

**FIG 5:**
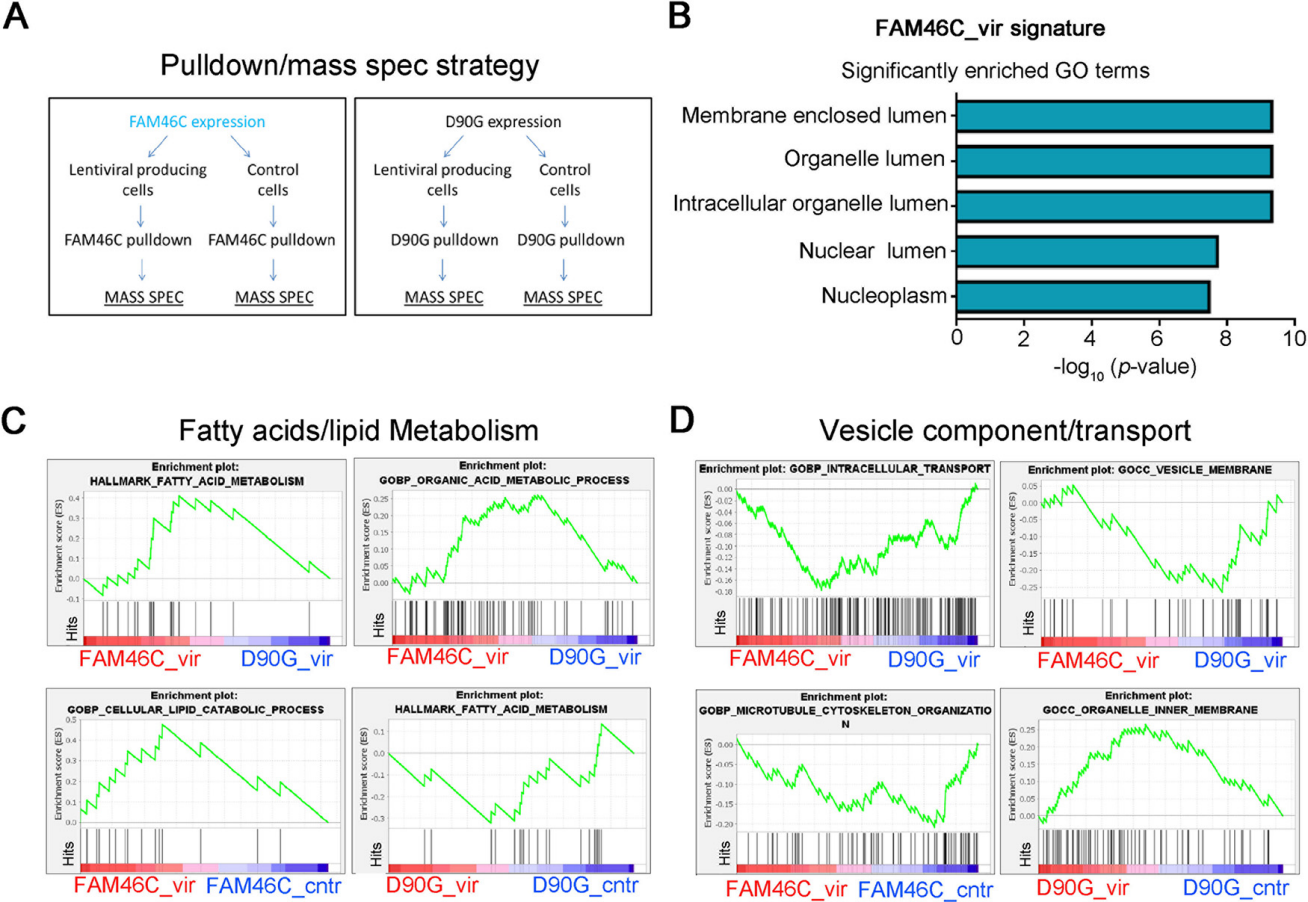
During lentiviral production, the interactome of FAM46C is enriched in proteins involved in organelle homeostasis and intracellular trafficking. (A) Cartoon depicting our pulldown/mass spectrometry approach. HEK-293T cells were transfected with plasmids encoding FAM46C or the D90G mutant either in the presence or in the absence of lentiviral packaging and envelope plasmids. FAM46C and D90G proteins were then coimmunoprecipitated, and their interactomes were analyzed by mass spectrometry. (B) GO terms significantly enriched for proteins that interact more with FAM46C during viral production. (C and D) GSEA on the interactomes of FAM46C and D90G during lentiviral production. By using GSEA, we compared the interactome of FAM46C with that of the D90G mutant during viral particle production (FAM46C_vir/D90G_vir) (top) and also each of the two interactomes with their counterpart in the absence of viral particle production (FAM46C_vir/FAM46C_cntr and D90G_vir/D90G_cntr) (bottom), highlighting fatty acid/lipid metabolism (C) and in vesicle component/transport (D) signatures.

To further dissect FAM46C mode of action in regulating viral replication, we took advantage of FAM46C vast proteome (6) and determined how it varied upon lentiviral particle production. By mass spectrometry, we analyzed the interactomes of both FAM46C and D90G in HEK-293T cells either left untreated or transfected with lentiviral envelope and packaging plasmids (Fig. 5A; see also Table S1 in the supplemental material). Recently, we have demonstrated that, in MM cells, the autophagic defects triggered by FAM46C are actually a consequence of altered intracellular trafficking (6); hence, we wanted to determine whether, during viral production in HEK-293T cells, there was an alteration of the FAM46C interactome that could pinpoint modulation of intracellular trafficking dynamics.

Among the top interactors enriched in the FAM46C interactome in the presence of viral proteins, we found MYH9, a cytoskeleton motor protein involved in intracellular trafficking and secretion, and PDIA3, an ER-resident protein involved in protein folding, whose localization is compatible with that of FAM46C (6) and with a role in intracellular trafficking regulation (see Table S1). Interaction with both proteins was confirmed by coimmunoprecipitation experiments (see Fig. S5A), strengthening the idea that FAM46C is regulating intracellular trafficking also in the scenario of viral protein production. By performing Gene Ontology (GO) analysis on the list of proteins differentially enriched during viral production in the FAM46C pulldown (see Table S1), we mostly found components of intracellular vesicles/organelles (Fig. 5B), suggesting that during viral replication FAM46C actually establishes an interactome which is connected to organelle homeostasis. To further explore our mass spectrometry data, we performed gene set enrichment analysis (GSEA) on the full list of protein interactors of FAM46C and of the D90G mutant during lentiviral particle production. We found a positive correlation with gene sets involved in fatty acid/lipid synthesis modulation (Fig. 5C, top; see also Fig. S5B, top) and a negative correlation with gene sets connected to intracellular transport and vesicle components (Fig. 5D, top; see also Fig. S5B, bottom). These results are in line with FAM46C being a regulator of intracellular vesicle/organelle homeostasis and trafficking dynamics. Further evidence was also obtained when we performed GSEA on the interactomes of either FAM46C or D90G during viral particle production and their respective counterparts in the absence of viral particle production.

For FAM46C, in fact, we found correlation with lipid/fatty acids metabolism (Fig. 5C, bottom; see also Fig. S5C, top) and inverse correlation with components of the cytoskeleton machinery and vesicle trafficking (Fig. 5D, bottom; see also Fig. S5C, bottom), while for the D90G we found an inverse correlation with fatty acid metabolism (Fig. 5C, bottom) and a positive correlation with components of intracellular vesicles (Fig. 5D, bottom). These results indicate that FAM46C might have a role in regulating intracellular trafficking also during viral production in HEK-293T cells, an event that could cause defective autophagy.

### FAM46C is stimulated by both type I and type II IFNs.

A first layer of response to viruses relies on innate immunity triggered by IFN responses. IFN receptor recognition triggers a myriad of intracellular signaling pathways and induces expression of a wide range of ISGs aimed at counteracting viral replication/particle production/release (15, 31). Previous high-throughput studies suggested FAM46C to be an ISG (15, 18), but this was never validated. Hence, we wanted to determine whether FAM46C expression could be actually modulated downstream of IFN signaling.

**FIG 6:**
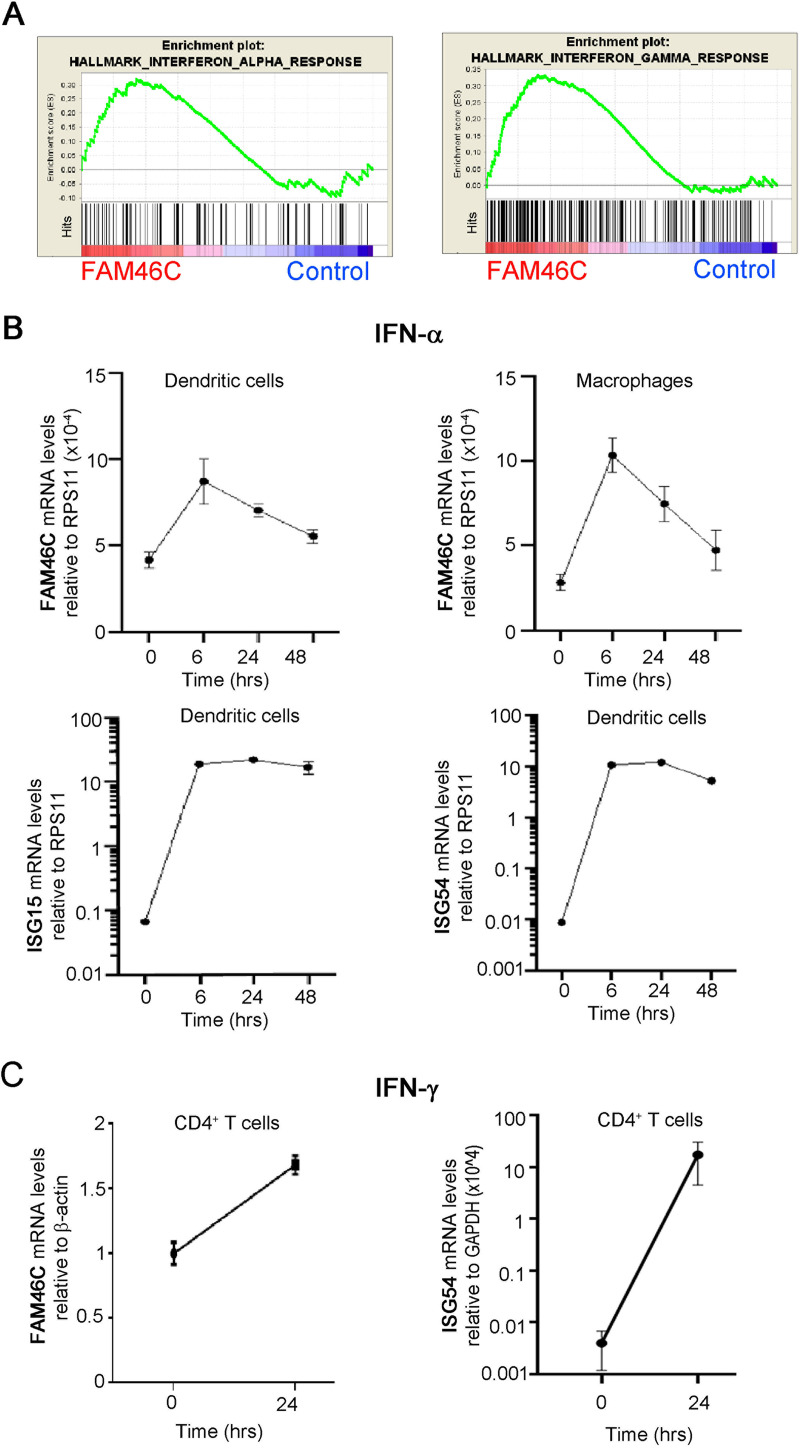
FAM46C is stimulated by both type I and type II IFNs. (A) GSEA on microarray data from OPM2 MM cells expressing either FAM46C or a mock control. Signatures related to IFN responses are shown. (B) FAM46C expression levels upon IFN-α administration. Dendritic cells and macrophages were isolated from the peripheral blood of healthy donors and stimulated with IFN-α for 48 h. At the time points indicated, the cells were harvested, and RNA was extracted. Transcript levels were determined by RT-qPCR with normalization on RPS11. ISG15 and ISG54 transcript levels were used as controls. (C) FAM46C expression levels upon IFN-γ administration. CD4^+^ T cells were isolated as described for panel B and stimulated for 24 h with IFN-γ. Transcript levels were determined by RT-qPCR with normalization on β-actin. ISG54 transcript levels were used as a control. Graphs represent the means ± the SD of three independent experiments.

At first, to obtain confirmation of a role for FAM46C in IFN responses we data-mined our previously published transcriptomic data from MM cells (6) (ArrayExpress accession number E-MTAB-8188). By GSEA, we found that FAM46C expression actually correlated with both IFN-α and IFN-γ gene expression signatures (Fig. 6A). Next, we tested whether FAM46C expression was actually IFN stimulated. At first, we analyzed FAM46C expression after type I IFN stimulation. We isolated CD14-expressing cells from peripheral blood mononuclear cells (PBMCs) of healthy donors and differentiated them *in vitro* in monocyte derived dendritic cells (MDDCs) or macrophages, the two major players involved in type I IFN response, and stimulated them *in vitro* with IFN-α. We monitored over time FAM46C mRNA levels through RT-qPCR and, in parallel, we analyzed transcript levels of well-known ISGs, namely, ISG15, ISG54, OAS1, and IFIT1. We found that in both cell types FAM46C mRNA levels nearly doubled after 6 h of IFN-α administration, while those of the other ISGs increased more drastically (10- to 1,000-fold) (Fig. 6B; see also Fig. S6A). Moreover, after 48 h of IFN-α administration, the expression of FAM46C dropped to basal levels, while that of the other canonical ISG mRNAs remained high. These results indicate that FAM46C expression can indeed be stimulated by type I IFNs but that FAM46C is a weak type I IFN-stimulated gene.

In addition to type I IFNs, type II IFNs are also important for orchestrating viral responses in immune cells. Specifically, during HIV infection high levels of IFN-γ are secreted by CD4^+^ and CD8^+^ T cells in order to support host defense (32). By analyzing previously published transcriptome sequencing (RNA-Seq) data (33), we found that FAM46C is highly present in all types of T-cell subpopulations, with the highest expression being found in CD4^+^ T cells (see Fig. S6B). Since CD4^+^ T cells are the immune subtype preferentially infected by HIV, we tested whether FAM46C expression could be induced by type II IFN (IFN-γ) in CD4^+^ T cells. We purified CD4^+^ T cells from the PBMCs of healthy donors and treated them *in vitro* for 24 h with 50 ng/mL of IFN-γ. Transcription of FAM46C was enhanced 2-fold by IFN-γ treatment (Fig. 6C), at least in part mimicking FAM46C response to IFN-α, suggesting that FAM46C is an ISG responding weakly also to type II IFNs.

In order to evaluate whether FAM46C expression could *per se* regulate the IFN signaling cascade, we checked for transcript levels of well-known IFN-stimulated transcription factors, namely, IRF9, STAT1, and STAT2, in cells overexpressing wt-FAM46C (26) (see Fig. S6C). Upon FAM46C overexpression, we found no significant difference in the abundance of all the transcripts analyzed, suggesting that FAM46C is not an upstream regulator but mainly a downstream target of the IFN pathway. In conclusion, our study demonstrates that FAM46C is a type I and type II ISG which inhibits viral spread by regulating autophagy and, possibly, intracellular trafficking dynamics.

## DISCUSSION

Here, we showed that FAM46C expression inhibits lentiviral particle production in HEK-293T cells both in *cis* and in *trans*. We demonstrated that the mechanism through which FAM46C inhibits viral production does not involve either regulation of the viral egress pathway, Gag-p55 protein processing, or alteration of viral mRNA levels but rather relies on the capacity of FAM46C to dampen the autophagic flux, a process that we found to be fundamental for efficient viral particle production in HEK-293T cells. In addition, we also showed that FAM46C gene expression is mildly induced by type 1 and type 2 IFNs.

We suggest that the capability of FAM46C to inhibit lentiviral production is, at least in part, related to its function as a tumor suppressor in MM cells, where its expression triggers apoptosis by dampening autophagic clearing of protein aggregates (6). In MM, FAM46C-induced autophagic inhibition is coupled with an alteration of intracellular trafficking and secretion (6), which has been proposed to be the cause of such autophagy defect. Consistently, the D90G mutant, that loses its tumor suppressor capability, neither impairs autophagy nor alters intracellular trafficking/secretion.

In the lentiviral particle production context, in HEK-293T cells, the D90G mutant has a relevant but not complete loss-of-function phenotype compared to wt-FAM46C, suggesting that the overall mechanisms, despite being shared, might not actually be completely overlappable or that cell-intrinsic factors might enter the picture. In line with this idea, we found that other FAM46C loss-of-function mutants, namely, the F184L and Y291C alleles (6), also negatively affected FAM46C capability to inhibit viral replication, but to different extents. Specifically, while the F184L substitution had a significant, but partial effect, the Y291C substitution fully restored the cells’ ability to produce lentiviral particles (see Fig. S1), indicating that different FAM46C loss-of-function mutant alleles actually behave slightly differently in the lentivirus-producing scenario but further strengthening the idea that a fully functional FAM46C is required for efficient inhibition of lentiviral particle production.

The interactome of FAM46C was modulated during viral production, being enriched in components of the lipid/fatty acid biosynthesis machinery and in motor proteins. The most enriched protein in the FAM46C pulldown during viral production was Myosin-9 (MYH9) (see Table S1 and Fig. S5A) a protein that is downmodulated by HIV in glomeruli (34) and is involved in both cytoskeleton organization and protein secretion (35). These observations, together with the inhibitory effect on autophagy, indicate that alteration of intracellular trafficking dynamics might be the cause of autophagic inhibition during viral production in wt-FAM46C expressing cells.

The molecular level at which autophagy plays a role for viral production in HEK-293T cells is unclear, but its relevance is not surprising. For instance, several studies indicated that autophagy, and specifically the autophagosomes, can function as a protective scaffold for viral particle production (22, 36–38). Picornaviruses and HIV use the double membrane of autophagosomes as a scaffold for RNA assembly and replication (39, 40), while hepatitis C virus seems to rely on autophagy not only for replication but also for efficient translation (41). We did not observe major differences in the levels of viral transcripts or in the efficiency of their translation, suggesting that autophagosomes may favor viral production by preserving the integrity of viral proteins. Viral proteins have long been known to be targeted for degradation by the intracellular polyubiquitination system. One hypothesis could be that autophagosomes are important for “hiding” viral proteins from degradation by the proteasome, an assumption that is corroborated by the fact that proteasomal inhibition augments intracellular viral protein levels (see Fig. S4F). In this light, autophagy was shown to be important for foot-and-mouth disease virus (FMDV) replication, and FMDV-infected cells showed colocalization of viral proteins with autophagosomal markers (42). Similarly, autophagy was shown to be important in early stages of HIV-1 replication in macrophages, where LC3 colocalized, copurified, and coprecipitated with the Gag protein (40). However, future studies are required to confirm this working model.

The fact that FAM46C was picked up in several different screenings for detecting ISGs using different cell lines and different virus types indicates that FAM46C regulatory role on viral replication might be broader and possibly more complex that what one might think.

In line with this idea, when we checked the effect of FAM46C downmodulation on the replication of the HIV-1 NL4-3 nef-IRES GFP clone in a CD4^+^-derived cell line, we found an increase in viral particle replication, confirming the inhibitory effect of FAM46C. Interestingly, however, the effect was not as strong as the one seen during lentiviral particle production in HEK-293T cells, suggesting that either accessory viral proteins might be capable to counteract FAM46C inhibitory function in specific contexts or that certain cell types, such as CD4^+^ T cells in this specific case, could be less dependent on autophagy for efficient viral particle production and consequently less sensitive to alterations in FAM46C expression. Future studies will be required to validate these hypotheses.

Recently, FAM46C was found as a hit in a screening to determine SARS-CoV-2 antiviral genes. Intriguingly, in this study, FAM46C was shown to inhibit viral entry (43). This might suggest that either FAM46C can alter intracellular pathways other than autophagy or that autophagy can, by some means, regulate also this specific stage of the viral life cycle, as previously proposed (44). Notably, in a different cell context and with different viruses, FAM46C exerts opposite functions, either antiviral or proviral (15, 17, 18), suggesting that FAM46C is not *per se* an antiviral protein. More broadly, we can speculate that the physiological role of FAM46C is linked to the regulation of intracellular trafficking dynamics and secretion and that the downstream secondary effects induced by such modulation can vary between cell types and conditions analyzed and might have drastically different outcomes. Indeed, for both the tumor suppressor and antiviral functions of FAM46C, the relevant downstream phenotypes always come as indirect secondary effects: in MM, where there is high ER stress due to the highly secretory nature of plasma cells, unfolded proteins accumulate and autophagic dampening causes defective protein clearing, in turn causing apoptosis, while in HEK-293T cells, where autophagy is required for efficient viral replication, autophagic inhibition causes defective viral particle production. In line with this view, (i) FAM46C expression does not directly affect viral particle egress, indicating that viral particles are not the direct targets of FAM46C-regulated secretion, and (ii) FAM46C is not a canonical IFN stimulated gene but only a weak responder to IFN stimulation, underlining that viral inhibition is not FAM46C primary physiological function. It would be interesting to determine whether, in contexts in which FAM46C behaves as a proviral factor, the effect is still exerted through autophagic inhibition or if different intracellular pathways are involved.

FAM46C is part of a high-molecular-weight complex which resides at the ER (6, 8), and its localization requires interaction with Fibronectin type 3 domain protein 3A, FNDC3A. In MM FAM46C-induced phenotypes require expression of FNDC3A (6). It would be interesting to test whether FAM46C antiviral function requires its localization at the ER and/or its interaction with the FND3A protein. Intriguingly, FNDC3B, a paralog of FNDC3A which also interacts with FAM46C (8), was shown to be a target of HBV and was proposed to function as an antiviral factor (45), suggesting an actual involvement of FNDC proteins in viral replication.

FAM46C is part of a protein family comprising three other proteins: FAM46A, FAM46B, and FAM46D. The four proteins share high similarity (6) but are differentially expressed among different cell types and tissues. FAM46C is highly expressed in B cells, CD4^+^ T cells (see Fig. S6B) and sperm cells (26), FAM46A is expressed mostly in osteoblasts (46) and glial cells (47), FAM46B is essential in embryonic stem cells (48), while FAM46D in present in testis germ cells (49). It would be interesting to test whether in different cellular context also the other members of the FAM46 family are capable to regulate viral replication and, if so, if this is performed through autophagic regulation or through other mechanisms, e.g., the proposed capability of FAM46 proteins to polyadenylate mRNA poly(A) tails (26). It is tempting to speculate that in different tissues different FAM46 proteins may regulate viral replication by different means.

**FIG 7:**
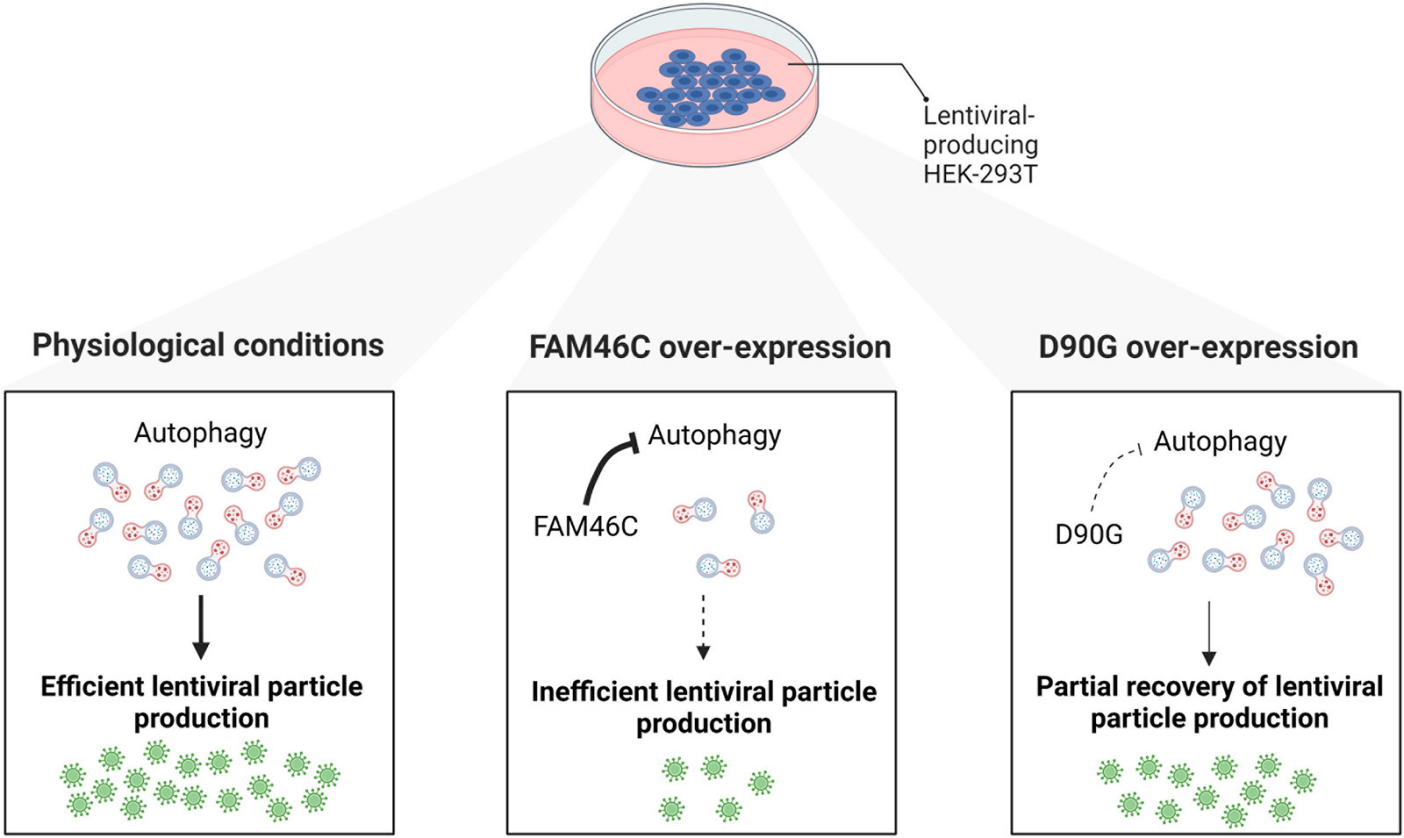
Cartoon depicting our working model. FAM46C expression inhibits lentiviral particle production by negatively regulating autophagy. (Left) In HEK-293T cells, the physiological levels of autophagy favor lentiviral replication and particle production. (Center) When FAM46C is overexpressed autophagy is dampened and virus replication and particle production are consequently reduced. (Right) The expression of the D90G mutant allele partially suppresses FAM46C-induced autophagy-dependent inhibition of viral replication and particle production.

In summary, oncosuppressor FAM46C is also an IFN-stimulated gene capable to interfere with efficient lentiviral particle production in HEK-293T cells, doing so by inhibiting autophagy (Fig. 7). We propose that the mode of action of FAM46C during lentiviral particle production is the same exerted during apoptosis induction in MM cells and relies on FAM46C capability to regulate intracellular trafficking dynamics. In this scenario, FAM46C’s drastic effect on viral production could be exploited as an output to rapidly screen for inhibitors or stimulators of FAM46C functionality, with a valuable impact for cancer therapy development.

## MATERIALS AND METHODS

### Cell culture and processing of PBMCs.

HEK-293T packaging cells were purchased from the American Type Culture Collection (ATCC). HEK-293T packaging cells were cultured in Dulbecco modified Eagle medium (DMEM) supplemented with 10% fetal bovine serum (FBS), 100 U/mL penicillin, 100 μg/mL streptomycin, and 1% glutamine.

HEK-293T Scramble and ATG5 sh cell lines were produced by transducing HEK-293T cells with lentiviruses expressing either a control vector or a vector harboring an shRNA targeting ATG5 (see “Lentiviral particle production” below). Stable cell lines were established after 4 days of puromycin selection at a concentration of 2 μg/mL and after testing ATG5 downmodulation through RT-qPCR (see “mRNA extraction and RT-qPCR”). The OPM2 MM cell line was kindly provided by G. Tonon (San Raffaele Scientific Institute, Milan, Italy). OPM2 cells were grown and maintained in RPMI 1640 containing 100 U/mL penicillin and 100 μg/mL streptomycin supplemented with 10% FBS (Gibco) and 1% glutamine. Cells were tested monthly for mycoplasma contamination. Stably silenced SUP-T1 cell lines were generated by transduction with lentiviral vectors encoding FAM46C-targeting shRNAs or nontargeting shRNAs (see “Lentiviral particle production”), followed by puromycin selection (1 μg/mL).

Buffy coats of healthy donors were obtained from Fondazione IRCCS Ca’ Granda Ospedale Maggiore Policlinico, Milan, Italy. PBMCs were isolated by centrifugation through a Ficoll-Paque density gradient. The IRCCS Ca’ Granda Ospedale Maggiore Policlinico Foundation ethical committee approved the use of PBMCs from healthy donors for research purposes, and all methods were performed in accordance with relevant guidelines and regulations. Informed consent was obtained from all donors. Human blood primary CD4^+^ T cells were purified >95% by negative selection with the isolation kit for human CD4^+^ T cells (Miltenyi Biotec). Human CD14^+^ cells were obtained from PBMCs by positive selection using CD14 MicroBeads (Miltenyi Biotec).

CD4^+^ cells were cultured *in vitro* in RPMI medium supplemented with 10% FBS, 0.1% penicillin-streptomycin (EuroClone), 1% glutamine, 0.1% nonessential amino acids (Lonza), and 0.1% sodium pyruvate (Lonza) at 37°C and 5% CO_2_. Cells were then treated with or without 50 ng/mL of IFN-γ for 24 h.

CD14^+^ cells were differentiated into MDDCs or macrophages. MDDCs were obtained by adding 20 ng/mL of human granulocyte-macrophage colony-stimulating factor (GM-CSF; Miltenyi Biotec) and 50 ng/mL human IL-4 (Miltenyi Biotec) to the culture medium for 5 to 6 days. Macrophages were obtained by culturing CD14^+^ cells with 50 ng/mL human GM-CSF for 10 days. The GM-CSF-containing medium was replenished at days 2, 5, and 8 of culture. MDDCs and macrophages were stimulated with 10 ng/mL of IFN-α. Cells were collected before stimulation (time zero) and at 6, 24, and 48 h poststimulation for RNA analysis.

### Plasmids.

The human FAM46C gene was subcloned from the nonlentiviral PCMV6-Entry FAM46C plasmid (Myc-DDK-tagged; Origene, RC215475) to the pLenti-C-Myc-DDK expression vector (Origene, PS100064) through insertion in the Sgf1 and MluI sites, giving rise to the pLenti-FAM46C-C-Myc-DDK plasmid. For doxycycline-inducible plasmids, the full-length FAM46C sequence was PCR amplified from the PCMV6-Myc-DDK FAM46C plasmid (Origene, RC215475) and ligated into the NheI and BamHI sites of doxycycline-inducible pcW lentiviral expression vector (Addgene) as described previously (6), giving rise to the pCW-FAM46C-C-Myc-DDK. The D90G, F184L, and Y291C FAM46C missense point mutant variants were generated using a QuikChange II site-directed mutagenesis kit (Agilent Technologies) as previously described (6). The GFP vector was obtained by GFP cloning into the transfer vector pCCL-PPT-hPGK, as described previously (50).

### Lentiviral particle production.

HEK-293T packaging cells were transiently transfected through calcium phosphate precipitation with envelope plasmid VSV-G, packaging plasmids PMDLg/pRRE and pREV, and one of the following transfer vectors: pLenti-FAM46C-C-Myc-DDK, pLenti-D90G-C-Myc-DDK, pLenti-Y291C-C-Myc-DDK, pLenti-F184L-C-Myc-DDK, pCCL-PPT-hPGK-GFP, pCW-FAM46C-C-Myc-DDK, pCW-D90G-C-Myc-DDK, MISSION pLKO.1-puro Non-Target shRNA Control, MISSION pLKO.1-puro sh-ATG5 (Sigma-Aldrich, clone TRCN0000151963) or MISSION pLKO.1-puro sh-FAM46C (Sigma-Aldrich, clone TRCN0000166958). At 24 h after transfection, exhausted medium was removed, and fresh medium was added. Virus-containing supernatants were collected 48 h after transfection, filtered through 0.45-μm-pore size filters (Millipore), and kept at −80°C until use. For GFP-expressing lentiviral particle production experiments, HEK-293T cells were also cotransfected with PCMV6-FAM46C or PCMV6-D90G nonlentiviral plasmids.

For rapamycin experiments GFP-expressing lentiviruses were collected at 48 h after a 24-h treatment with 100 nM rapamycin. For BTZ experiments GFP-expressing cells were collected at 48 h after a 4-h treatment with 1.5 μM BTZ. For production of HIV-1 clones, the following plasmids were used: pZM247FV2, pZM247FV1, pTHROc./2626, pRHPAc./2635, HIV R7/3-EGFP, and HIV NL4.3. pZM247FV2, pZM247FV1, pTHROc./2626, and pRHPAc./2635 were kindly provided by Frank Kirchhoff, Institute of Molecular Virology, Ulm University Medical Center (51). HIV R7/3-EGFP was kindly provided by Cecilia Cheng Mayer, Aaron Diamond AIDS Research Center, The Rockefeller University, New York, NY (52), while HIV NL4.3 was obtained from the AIDS Research and Reference Reagent Program (53).

### Production of stable HEK-293T cell lines with inducible FAM46C and D90G constructs.

Stable HEK-293T cell lines carrying either inducible wt-FAM46C or inducible D90G constructs were generated through viral transduction with viruses carrying the pCW-FAM46C-C-Myc-DDK and pCW-D90G-C-Myc-DDK vectors. After 24 h of viral transduction, cells with integrated constructs were selected using 1 μg/mL puromycin (Sigma-Aldrich). Expression of FAM46C or of the D90G mutant allele was induced with 1 μg/mL doxycycline.

### Viral titer.

Viral titers were measured by using a Lenti-X p24 Rapid Titer kit (Clontech) according to the manufacturer’s instructions.

### FAM46C or D90G lentiviral transduction of OPM2 MM cells.

One million OPM2 cells were transduced with 1 mL of crude viral supernatant containing either the pLenti-FAM46C-C-Myc-DDK or pLenti-D90G-C-Myc-DDK transfer vector in the presence of 8 μg/mL Polybrene. After centrifugation at 500 × *g* for 50 min at room temperature, the cells were incubated at 37°C in 5% CO_2_ for 12 h. After a medium switch, the cells were cultured for another 60 h to assess, by Western blotting, FAM46C and D90G protein expression.

### GFP lentiviral vector transduction of HEK-293T cells and analysis of GFP^+^ cells.

HEK-293T cells (7.5 × 10^4^ cells/well) were seeded into 6-well plates and, at 24 h postplating, 0.2 mL of cell-free supernatant containing GFP lentiviral particles produced in cells expressing either wt-FAM46C or the D90G mutant allele were added to each well. Then, 8 μg/mL Polybrene was added. At 24 h postransduction, media were replaced with fresh complete DMEM. The cells were then collected at either day 5 (for cotransfection experiments) or day 10 (for experiments using inducible vectors) postransduction for detection of the GFP^+^ subpopulation. GFP^+^ cells were detected both by flow cytometry and fluorescence microscopy. For flow cytometry analyses, cells were washed twice in phosphate-buffered saline (PBS), and analyzed for GFP expression by using a BD FACS CANTO II flow cytometer; analyses were performed using the BD FACSDIVA software. For fluorescence microscopy analyses, cells plated in 24 multiwell plates were washed three times in PBS and then fixed in 2% paraformaldehyde (PFA) for 10 min at room temperature. After three subsequent washes in PBS, the cells were analyzed using a Nikon-Crest microscope. The number of GFP^+^ cells was determined using Fiji software.

For rapamycin experiments, the same procedures were followed except that the supernatants were obtained from cells treated for 24 h with 100 nM rapamycin.

### SUP-T1 infection with HIV clone pBR HIV-1 NL4-3 nef-IRES GFP.

The replication-competent HIV molecular clone pBR HIV-1 NL4-3 nef-IRES GFP was kindly provided by Frank Kirchhoff, Institute of Virology, University of Ulm, and was previously described (25). Viral stocks were obtained by transfecting HEK-293TN cells with the corresponding plasmid using the calcium phosphate technique. Viral stocks were concentrated by ultracentrifugation at 80,000 × *g* on Beckman ultracentrifuge using an SW32Ti swinging rotor for 2 h. Pellets were resuspended in 1:100 of the initial volume in PBS, aliquoted, and stored at −80°C. The viral stock concentration was assessed by using an HIV combo antigen-antibody enzyme-linked immunosorbent assay (ELISA; Dia.pro Diagnostic BioProbes).

SUP-T1 cells (0.5 × 10^6^; transduced with FAM46C shRNA or NT shRNA) were infected overnight (with 250 ng of p24) in a 96-well format in the presence of Polybrene at 2 μg/mL. At 4 days postinfection, the cells were fixed with 4% PFA, and the GFP expression was monitored by flow cytometry using a FACSCanto II machine. Data were analyzed using BD FACSDIVA and FlowJo softwares.

### Western blotting and antibodies.

SDS-PAGE, followed by Western blotting, was performed on protein extracts derived from (i) OPM2 cells transduced with viruses expressing either wt-FAM46C or the D90G mutant allele; (ii) HEK-293T cells transfected with plasmids encoding for wt-FAM46C or the D90G, Y291C, and F184L mutant alleles, along with the envelope VSV-G and PMDLg/pRRE and pREV packaging plasmids; or (iii) trichloroacetic acid-precipitated supernatants of HEK-293T virus-producing cells. Cells were collected and lysed in radioimmunoprecipitation assay buffer (10 mM Tris-HCl [pH 7.4], 1% sodium deoxycholate, 1% Triton X-100, 0.1% SDS, 150 mM NaCl, and 1 mM EDTA [pH 8.0]) at 48 h postransduction or transfection, respectively. Supernatants were collected at 48 h posttransfection. Viral particle proteins were detected using the serum of an HIV-1 individual containing high titers of α-HIV-1 antibodies at a 1:1,000 dilution and secondary HRP-goat anti-human IgG (H+L) antibodies (1:5,000 dilution; Thermo Fisher Scientific, catalog no. 81-7120).

Processing of Gag p55 and Gag p24 levels were determined using α-HIV-1 p24 antibodies (1:2,000 dilution; Abcam, catalog no. 9071), and FAM46C levels were determined using α-FLAG antibodies (1:1,000 dilution; Sigma-Aldrich, catalog no. F1804). Autophagic markers p62 and LC3 were detected using α-p62 (1:1,000 dilution; Cell Signaling, catalog no. 88588) and α-LC3 (1:1,000 dilution; Cell Signaling, catalog no. 2775) antibodies. For BTZ experiments, the accumulation of polyubiquitinated proteins was assessed using α-polyubiquitin antibodies (1:1,000 dilution; Cell Signaling, catalog no. 3933). β-Actin and GAPDH were detected using α-β-actin (1:4,000 dilution; Sigma-Aldrich, catalog no. A5441) and α-GAPDH (1:1,000 dilution; Cell Signaling, catalog no. 2118) antibodies.

Western blot signals were detected using Super Signal West Pico Plus chemiluminescent substrate (Thermo Fisher Scientific, catalog no. 34577), according to the manufacturer’s instructions, and an iBright imaging system (Thermo Fisher Scientific).

### Apoptosis assay.

Apoptosis analyses were performed after transfection of HEK-293T cells with envelope and the packaging plasmids VSV-G, PMDLg/pRRE, and pREV, along with the transfer vectors pLenti-FAM46C-C-Myc-DDK and pLenti-D90G-C-Myc-DDK. Apoptosis was quantified using annexin V/propidium iodide double staining with a Dead Cell Apoptosis kit (Thermo Fisher Scientific, catalog no. V13241) as described previously (6). Samples were acquired using a FACSCanto II machine and analyzed with BD FACSDIVA and FlowJo softwares.

### mRNA extraction and RT-qPCR.

Total RNA from human CD4^+^ T lymphocytes, macrophages, dendritic cells, and HEK-293T virus-producing or SUP-T1 cells was extracted using TRIzol reagent (Thermo Fisher Scientific, catalog no. 15596026) and an RNeasy minikit (Qiagen, catalog no. 74104) as described previously (54). Next, 1 μg of total RNA was reverse transcribed using random primers and SuperScript III First-Strand Synthesis SuperMix (Invitrogen, catalog no. 11752-050). The synthesized cDNA was then used for RT-qPCR using either GoTaq qPCR Master Mix (Promega, catalog no. A6001) or TaqMan Universal PCR Master Mix (Thermo Fisher Scientific, catalog no. 4304437) on a StepOnePlus system (Thermo Fisher Scientific).

The following primers were used to detect FAM46C and ISG levels in CD4^+^ T cells, macrophages, and dendritic cells using GoTaq qPCR master mix: β-Actin FWD, AGAGCTACGAGCTGCCTGAC; β-Actin REV, CGTGGATGCCACAGGACT; FAM46C Endo FWD, GTCGCCTCCTCTTCTATTGC; FAM46C Endo REV, GACATGCAATCCCTGGTACA; OAS1 FWD, GATCTCAGAAATACCCCAGCCA; OAS1 REV, AGCTACCTCGGAAGCACCTT; IFIT1 FWD, AGTGTGGGAATACACAACCTACT; IFIT1 REV, GGTCACCAGACTCCTCACATTT; ISG54 FWD, ATGTGCAACCTACTGGCCTAT; ISG54 REV, TGAGAGTCGGCCCATGTGATA; ISG15 FWD, TCCTGGTGAGGAATAACAAGGG; ISG15 REV, GTCAGCCAGAACAGGTCGTC; RSP11 FWD, GCCGAGACTATCTGCACTAC; and RSP11 REV, ATGTCCAGCCTCAGAACTTC.

GAG mRNA detection in virus-producing HEK-293T cells was assessed using a TaqMan Universal PCR Master Mix (Applied Biosystems, catalog no. 4324018) and the following primers and probe: GAG FWD, 5′-ACATCAAGCAGCCATGCAAAT-3′; GAG REV, 5′-ATCTGGCCTGGTGCAATAGG-3′; and GAG PROBE, 5′ (FAM)-CATCAATGAGGAAGCTGCAGGAATGGGATAGA (TAMRA)-3′ (55). 18S rRNA was used as an internal standard (Applied Biosystems, 4333760F). The thermal cycling conditions for RT-qPCR were as previously described (56). Data are expressed as percentages of the relative quantity of target genes.

ATG5 levels in HEK-293T scramble and sh-ATG5 cells were determined using TaqMan Universal PCR Master Mix (Applied Biosystems, catalog no. 4324018) and Hs00355494_m1 probe (Thermo Fisher Scientific, catalog no. 4331182). FAM46C levels in SUP-T1 scramble and sh-FAM46C cells were determined using TaqMan Universal PCR Master Mix (Applied Biosystems, catalog no. 4324018) and Hs01933465_s1 probe (Thermo Fisher Scientific, catalog no. 4331182). 18S rRNA was used as an internal standard (Applied Biosystems, catalog no. 4333760F).

### Polysome profiling and RNA precipitation from polysomal fractions.

Polysome profiles from virus-producing HEK-293T cells were prepared as described previously (57, 58). In brief, cells were lysed in 50 mM Tris-HCl (pH 7.5), 100 mM NaCl, 30 mM MgCl_2_, 0.1% Nonidet P-40, 100 μg/mL cycloheximide, and 40 U/mL RNasin (Promega, catalog no. N2111) for 30 s on in ice. Crude extracts were then clarified by using centrifugation at 14,000 rpm for 5 min at 4°C.

Cytoplasmic extracts containing equal amounts of RNA were loaded onto 15 to 50% sucrose density gradients and centrifuged at 4°C for 3 h 30 min at 39,000 rpm in an Optima XPN-90 ultracentrifuge using an SW41Ti rotor. The absorbance at 254 nm was recorded using a BioLogic LP machine (Bio-Rad), and 1-mL fractions were collected for subsequent RNA extraction.

### RNA extraction from polysomal fractions and subsequent RT-qPCR.

Sub-, light, and heavy polysome fractions were extracted from sucrose gradient aliquots, as described previously (59). Briefly samples were incubated with 100 μg/mL proteinase K and 1% SDS for 2 h at 37°C. RNA was then extracted according to the phenol-chloroform/isoamyl acid method. After RNA purification, samples were retrotranscribed using SuperScript III First-Strand Synthesis SuperMix (Invitrogen, catalog no. 11752-050), and the synthesized cDNA was then used for RT-qPCR with GoTaq qPCR Master Mix (Promega, catalog no. A6001) on a StepOnePlus system (Thermo Fisher Scientific). The following oligonucleotides were used: GAG_1_FWD, AGCGTCAGTATTAAGCGGGG; GAG_1_REV, CAGGCCAGGATTAACTGCGA; VSV_G_FWD, TATTGCCCGTCAAGCTCAGA; and VSV_G_REV, GACACATCCAACCGTCTGCT. The percentages of transcripts in sub-, light, and heavy polysome fractions were determined.

### Coimmunoprecipitation of the FAM46C and D90G proteins.

Coimmunoprecipitations were performed as described previously (6). Briefly, HEK-293T packaging cells were calcium phosphate transfected in 150-mm plates with either (i) packaging and envelope plasmids and FAM46C or D90G transfer vectors or (ii) FAM46C or D90G transfer vectors alone. At 24 h posttransfection, the medium was replaced. Coimmunoprecipitations were performed at 48 h posttransfection. Cells were harvested and lysed in lysis buffer (50 mM Tris-HCl [pH 7.4], 150 mM NaCl, 1% Triton, 1 mM MgCl_2_, and 1:100 protease inhibitor cocktail [Sigma, catalog no. 8340]). Then, 3-mg portions of total cell lysates were incubated for 4 h at 4°C in constant rotation with protein G-Dynabeads (Life Technologies, catalog no. 10004D), previously incubated with 1 mg/mL anti-FLAG antibodies (Sigma, catalog no. F1804). After three washes in lysis buffer, the samples were boiled in 2× Laemmli buffer and stored at −80°C until use.

### Mass spectrometry and data analysis.

Coimmunoprecipitation samples were thawed in ice and loaded on SDS-PAGE gels (NuPAGE Novex 4 to 12%), stained with Coomassie brilliant blue (Sigma-Aldrich), and five consecutive bands were excised and trypsin digested as described previously (60). Subsequently, peptides extracted from the gel pieces were cleaned up using homemade stage-tip microcolumns (61). Peptides were then eluted in 40 μL of buffer B (80% acetonitrile [ACN], 0.1% formic acid [FA]). ACN was evaporated using a SpeedVac concentrator (Eppendorf), and the volumes of the eluates were adjusted to 5 μL with 1% trifluoroacetic acid (TFA) and then analyzed by liquid chromatography-tandem mass spectrometry (LC-MS/MS) using an EASY-nLC 1200 (Thermo Fisher Scientific, catalog no. LC140) connected to a Q Exactive Plus instrument (Thermo Fisher Scientific) through a nanoelectrospray ion source (Easy-Spray; Thermo Fisher Scientific). The nano-LC system was operated in a one-column setup with an Easy-Spray PEPMAP RSLC C_18_ column (Thermo Fisher Scientific) kept at 45°C constant. Solvent A was 0.1% FA, and solvent B was 0.1% FA in 80% ACN. Samples were injected in aqueous 1% (TFA) at a constant pressure of 9.8E+7 Pa. Peptides were separated with a gradient of 5 to 30% solvent B over 35 min, followed by gradients of 20 to 30% for 5 min and 30 to 65% over 3 min at a flow rate of 300 nL/min. The MS instrument was operated in the data-dependent acquisition mode to automatically switch between full scan MS and MS/MS acquisition. MS spectra (from *m/z* 375 to 1,650) were analyzed in the Orbitrap detector with resolution *R* = 70,000 at *m/z* 400. The 12 most intense peptide ions with charge states of ≥2 were sequentially isolated to a target value of 3e6 and fragmented with a normalized collision energy setting of 28% in to the HCD cell. The maximum allowed ion accumulation times were 20 ms for full scans and 45 ms for MS/MS. The dynamic exclusion time was set to 20s. Acquired raw data were analyzed using MaxQuant version 1.6.2.3 integrated with Andromeda search engine (62). The false discovery rate of all peptide identifications was set to a maximum of 1%. Carbamidomethylation of cysteine was set as a fixed modification. Two UniProt databases were specified for the search: UP000005640 (human). The LFQ intensity calculation was enabled, as well as the “match between runs” feature (63). The “protein groups” output file from MaxQuant was analyzed using Perseus software (64). Briefly, no imputation was used, and the data were filtered to have 75% valid values in at least one group. The *t* test statistic was used to identify significant proteins (see Table S1 in the supplemental material).

### GO TERM analysis.

GO term analyses on mass spectrometry data were performed using the Generic Gene Ontology (GO) term finder online tool (https://go.princeton.edu/cgi-bin/GOTermFinder).

### GSEA on previously published microarray data derived from MM cells and on MS data.

The previously published microarray data derived from OPM2 MM cells expressing either wt-FAM46C or D90G mutant allele (ArrayExpress accession number E-MTAB-8188 [https://www.ebi.ac.uk/arrayexpress/experiments/E-MTAB-8188]) were analyzed as described previously (6). The newly produced MS data from HEK-293T cells expressing either FAM46C or D90G mutant variant in the presence or absence of lentiviral proteins (PRIDE accession no. PXD036818) were analyzed as described below.

According to the protein levels measured in mass spectrometry, for each comparison (FAM46C_vir/D90G_vir, FAM46C_vir/FAM46C_cntr, and D90G_vir/D90G_cntr), each protein of the interactome was ranked by the difference in the two conditions. For each of the obtained ranked lists a preranked GSEA was performed to evaluate the enrichment of gene sets belonging to H (Hallmark gene sets), C2 (curated gene sets), and C5 (ontology gene sets) human collections of the Molecular Signature Database. The analysis was performed with the GSEA application v4.1.0 setting using the following parameters: number of permutations, 1,000; collapse/remap to gene symbols, collapse; chip platform, Human_Gene_Symbol_with_Remapping_MSigDB.v2022.1.Hs.chip; enrichment statistic, classic; maximum size, 500; and minimum size, 15.

### Validation of top FAM46C interactors in the presence of viral proteins.

FAM46C coimmunoprecipitation samples, prepared as described in “Coimmunoprecipitation of the FAM46C and D90G proteins,” were analyzed by SDS-PAGE, followed by Western blotting, as described in “Western blotting and antibodies,” using α-MYH9 (1:1,000 dilution; BioLegend, catalog no. 909801) and α-PDIA3 (1:1,000 dilution; BioLegend, catalog no. 937302) antibodies.

### Differential expression analysis of MM cell lines overexpressing FAM46C.

The differential expression analysis was performed on the following data sets from the Gene Expression Omnibus (GEO) series GSE83772 (26): (i) GSM2218717, GSM2218718, GSM2218719, and GSM2218720—total RNA of H929 cells expressing FAM46C^wt^; (ii) GSM2218713, GSM2218714, GSM2218715, and GSM2218716—total RNA of H929 cells expressing FAM46C with inactivating mutations D90A and D92A (FAM46C^mut^); (iii) GSM2218724, GSM2218725, and GSM2218726—total RNA of SKMM1 cells expressing FAM46C^wt^; and (iv) GSM2218721, GSM2218722, and GSM2218723—total RNA of SKMM1 cells expressing FAM46C^mut^.

For both cell lines, the gene count files of FAM46C^wt^ and FAM46C^mut^ were merged to obtain raw count matrices. Differentially expressed genes of the comparison “wt” versus “mut” were identified by running DESeq2 (65) 1.38.0 with ashr shrinkage (66) of log_2_FC (|log_2_FC|>1, Wald test *P* < 0.01).

### Band intensity quantitation.

The intensity of Western blot bands was quantified using the ImageJ software. Quantitations were performed on at least *n* = 3 blot images.

### Statistical analysis.

Results are given as means ± the standard deviations (SD) or means ± the standard errors of the mean. Statistical *P* values, calculated using a two-tailed *t* test, are indicated by asterisks in the figures (*, *P* < 0.05; **, *P* < 0.01; ***, *P* < 0.001; ****, *P* < 0.0001).

### Data availability.

Data were deposited in the PRIDE proteomics database under accession number PXD036818.
