# Supplementary material for: FAM46C Is an Interferon-Stimulated Gene That Inhibits Lentiviral Particle Production by Modulating Autophagy
Source: Microbiol Spectr. 2023 Jun 26;11(4):e05211-22. doi: 10.1128/spectrum.05211-22 (PMC10434054; doi:10.1128/spectrum.05211-22)
Supplement: Supplemental file 1 — Supplemental material. Download spectrum.05211-22-s0001.pdf, PDF file, 2.8 MB [file spectrum.05211-22-s0001.pdf]

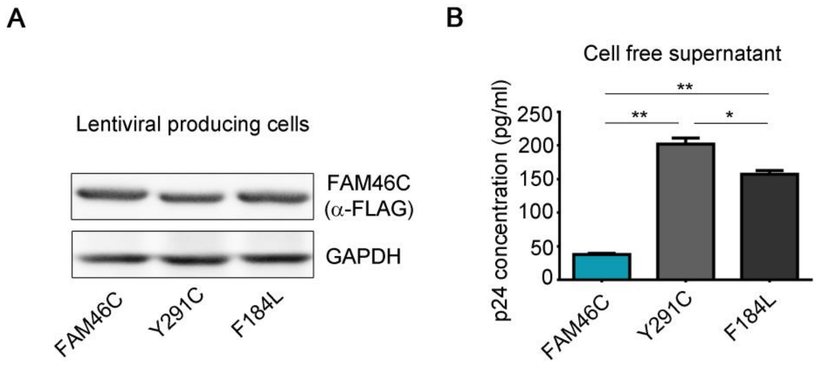

Fig.S1. Loss-of-function variants of FAM46C do not affect lentiviral particle production. Related to Fig.1.

(A) Western blot analysis showing protein levels of loss-of-function FAM46C variants in viral-producing HEK-293T cells. HEK-293T cells were transfected to produce lentiviral vectors expressing either FAM46C or the Y291C or F184L mutant variants as described in Fig. 1A. Samples were then prepared as described in Fig.1B. GAPDH was used as loading control.

(B) p24 concentrations in the lentiviral-enriched supernatants produced by HEK-293T cells. The lentiviral-containing supernatants produced by cells described in (A) were analysed for p24 levels by ELISA.

Histograms represent the means +/- SD of three independent experiments.

Statistical *P*-values were calculated using double-tailed unpaired *t* tests. **, $P<0.01$; ***, $P < 0.001$.

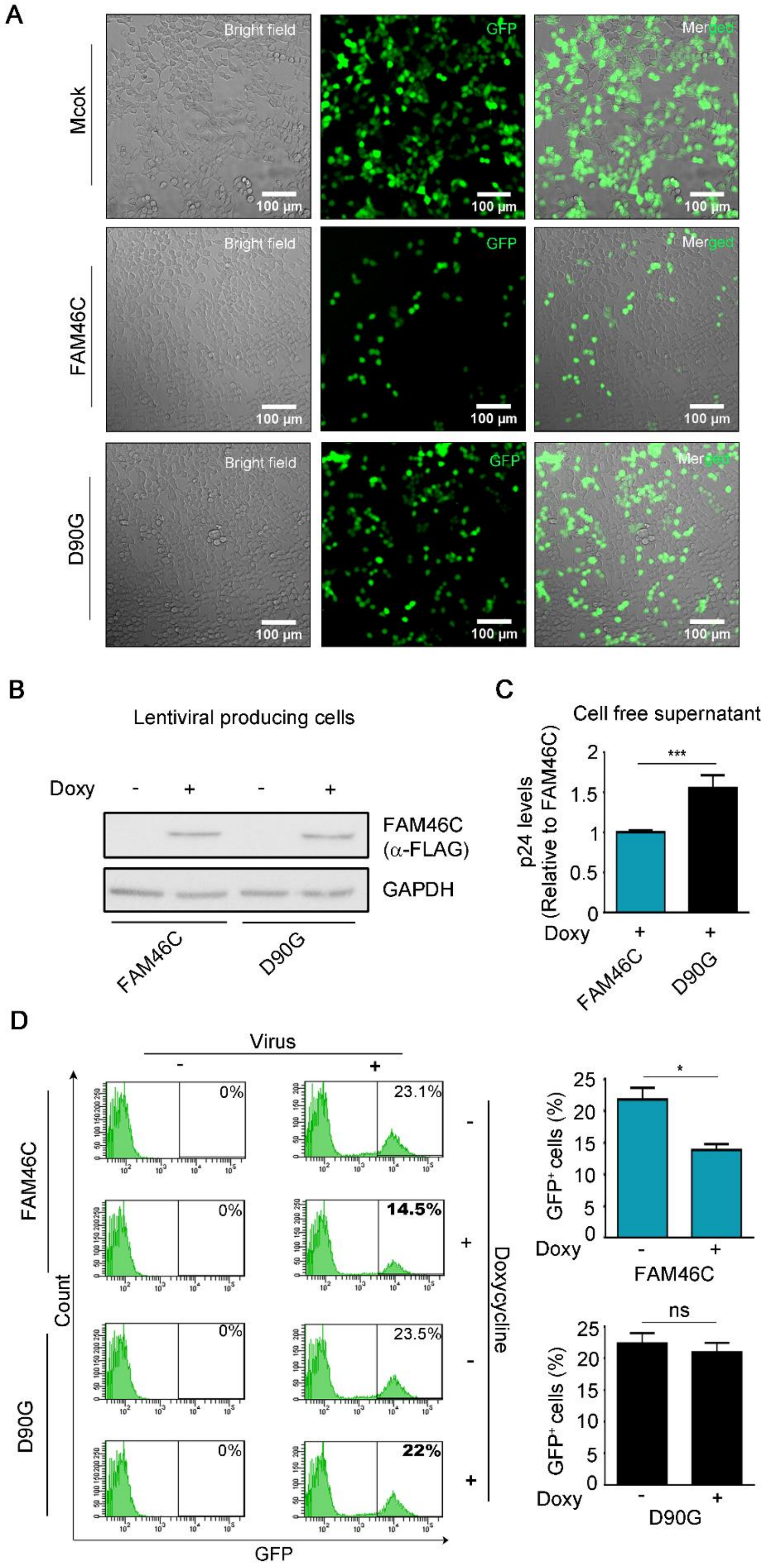

A
Mcok
FAM46C
D90G
Bright field
GFP
Merged
100 µm
B
Lentiviral producing cells
Doxy - + - +
FAM46C (α-FLAG)
GAPDH
FAM46C
D90G
C
Cell free supernatant
p24 levels (Relative to FAM46C)
***
Doxy + +
FAM46C
D90G
D
Virus
- +
0%
23.1%
14.5%
23.5%
22%
Count
GFP
Doxycycline
GFP+ cells (%)
*
ns
Doxy - +
FAM46C
D90G

Fig.S2. Triggering FAM46C expression in a genetically modified HEK-293T cell line inhibits lentiviral particle production. Related to Fig.2.

(A) Representative fluorescence microscopy images of HEK-293T cells re-infected with GFP-expressing lentiviruses produced in cells expressing either an empty vector (mock), FAM46C or the D90G mutant variant. Samples were prepared as in Fig. 2C.

(B-D) GFP lentiviral particles were produced in genetically engineered HEK-293T cell lines harbouring the FAM46C gene or the D90G mutant allele downstream of a doxycycline-inducible promoter either in the presence or absence of doxycycline. Viral-enriched supernatants were then used to infect fresh HEK-293T cells.

(B) Representative western blot of FAM46C and D90G protein levels in genetically engineered HEK-293T cell lines treated or not with Doxycycline.

(C) p24 concentrations in the lentiviral-enriched supernatants produced by genetically engineered HEK-293T cells treated with doxycycline. p24 levels were assessed by ELISA.

(D) (Left) Representative histogram showing the percentage of GFP^+ HEK-293T cells after transduction with GFP-expressing lentiviral particles produced in genetically engineered HEK-293T cells treated or not with doxycicline. (Right) Quantitation of the percentage of GFP^+ cells. Histograms represent means +/- SD of three independent experiments.

Statistical *P*-values were calculated using double-tailed unpaired *t* tests. ns, $P > 0.05$; *, < 0.05; ***, $P < 0.001$. Doxy: doxycycline.

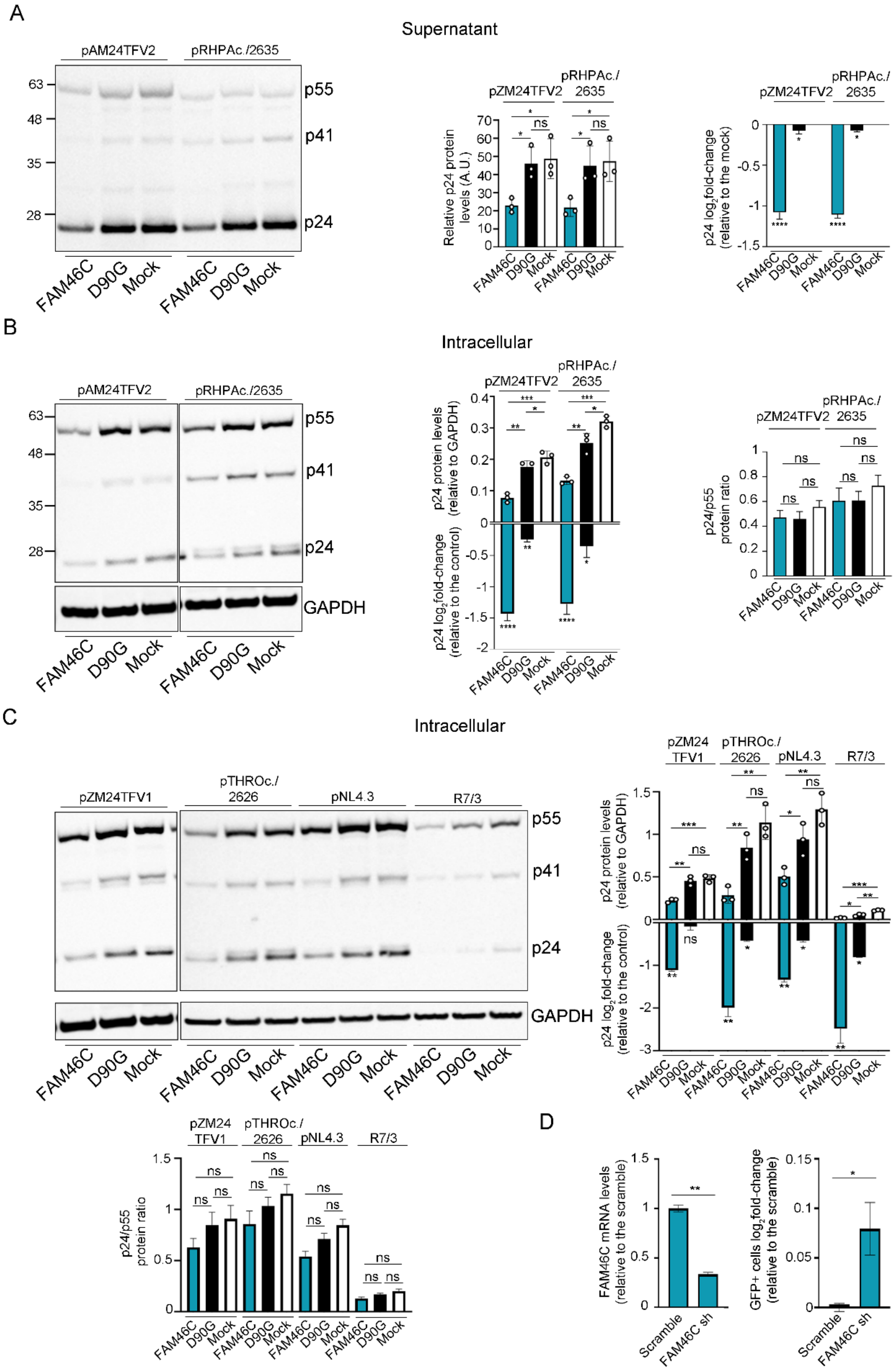

A
Supernatant
pAM24TFV2
pRHPAc./2635
p55
p41
p24
FAM46C
D90G
Mock
pZM24TFV2
pRHPAc./ 2635
Relative p24 protein levels (A.U.)
p24 log2fold-change (relative to the mock)
B
Intracellular
GAPDH
p24 protein levels (relative to GAPDH)
p24 log2fold-change (relative to the control)
p24/p55 protein ratio
C
Intracellular
pZM24TFV1
pTHROc./ 2626
pNL4.3
R7/3
pZM24 TFV1
D
FAM46C mRNA levels (relative to the scramble)
GFP+ cells log2fold-change (relative to the scramble)
Scramble
FAM46C sh

Fig. S3. FAM46C inhibits HIV-1 particle production and HIV-1 replication. Related to Fig.3.

(A) (Left) Representative western blot showing the levels of Gag viral proteins in the supernatant of HIV-1-producing HEK-293T cells. HEK-293T cells were transfected with plasmids in order to produce viral particles of the HIV-1 clones indicated in the presence of either and empty vector (Mock) FAM46C or the D90G mutant. Protein samples were obtained through TCA precipitation of supernatants and subjected to SDS-PAGE and western blotting using α–p24 antibodies. Same quantities of protein lysates were tested. (Right) Histograms representing quantitation of p24 band intensities and the relative $\log_2$ fold change of p24 band intensities between FAM46C-, D90G- and empty vector-expressing cells.

(B) (Left) Representative western blots showing the levels of Gag viral proteins in the cell lysates of HIV-1-producing HEK-293T cells. Same samples as in (A) were subjected to SDS-PAGE and western blotting to assess intracellular Gag protein levels using α–p24 antibodies. GAPDH was used as loading control. (Right) Histograms representing quantitation of p24 band intensities relative to GAPDH, relative $\log_2$ fold change of p24 band intensities between FAM46C-, D90G- and empty vector-expressing cells and p24/p55 band intensity ratios.

(C) (Top left) Representative western blots showing the levels of Gag viral proteins in the cell lysates of HEK-293T cells producing the HIV-1 clones indicated. Samples were prepared as described in (A). (Top right) p24 and (bottom) p24/p55 band quantitation as described in (B).

Blots are representative of three independent experiments.

(D) HIV-1 replication in SUP-T1 cells with FAM46C downmodulation

(Left) FAM46C downmodulation in SUP-T1 cells. SUP-T1 cells were transduced with lentiviral vectors expressing either a scramble control or an sh RNA targeting FAM46C. FAM46C downmodulation was confirmed through RT-qPCR. Histograms represent the means +/- SD of three independent experiments. (Right) Histograms representing the percentage of GFP^+ SUP-T1 cells after infection with pseudotyped GFP-wt-HIV-1 lentiviruses in the presence or absence of FAM46C downmodulation.

The $CD4^+$ SUP-T1 cells described above were infected with replication-competent pBR HIV-1 NL4-3 nef-IRES GFP viruses. 4-days post-infection viral replication was assessed by monitoring the percentage of GFP^+ cells through cytofluorimetric analysis. Histograms represent means +/- SEM, n=4. Statistical *P*-values were calculated using double-tailed unpaired *t* tests. ns, $P > 0.05$; *, < 0.05.

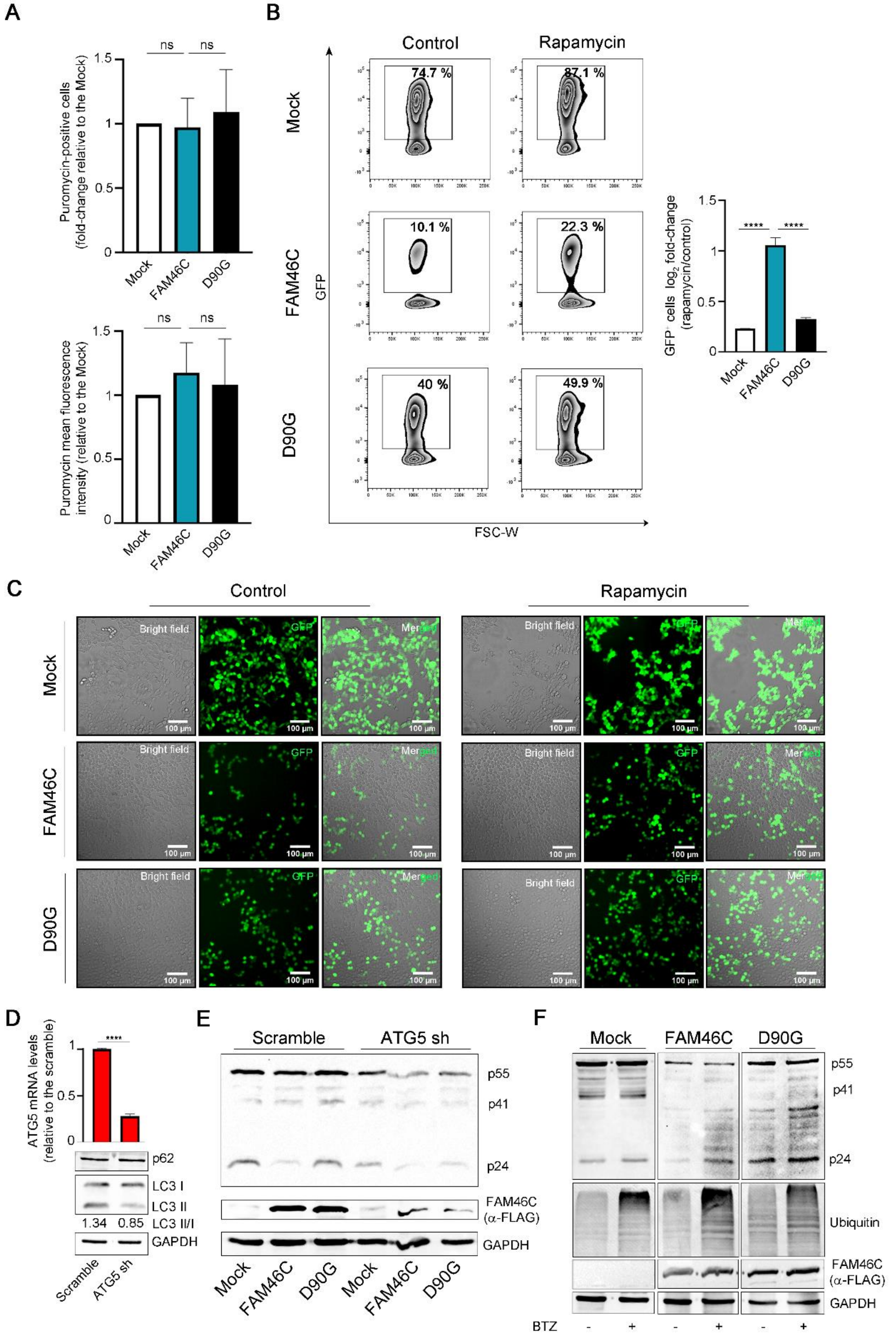

A
Puromycin-positive cells (fold-change relative to the Mock)
Puromycin mean fluorescence intensity (relative to the Mock)
ns
Mock
FAM46C
D90G
B
Control
Rapamycin
Mock
FAM46C
D90G
GFP
FSC-W
74.7 %
87.1 %
10.1 %
22.3 %
40 %
49.9 %
GFP⁺ cells log₂ fold-change (rapamycin/control)
****
C
Control
Rapamycin
Bright field
GFP
Merged
100 µm
D
ATG5 mRNA levels (relative to the scramble)
****
p62
LC3 I
LC3 II
1.34
0.85
LC3 II/I
GAPDH
Scramble
ATG5 sh
E
Scramble
ATG5 sh
p55
p41
p24
FAM46C (α-FLAG)
GAPDH
F
BTZ
Ubiquitin

Fig. S4. Autophagy is triggered during viral production in HEK-293T cells and rapamycin treatment restores the capability of FAM46C-expressing cells to efficiently produce lentiviral particles. Related to Fig. 4.

(A) Puromycin incorporation assay in HEK-293T cells transduced with lentiviral protein-expressing vectors. HEK-293T cells expressing either an empty vector, wt FAM46C or the D90G mutant allele were transfected with plasmids encoding for the lentiviral GAG/POL, VSV-G and REV genes and GFP. Forty-eight-hours post-transfection cells were treated with puromycin and assessed for puromycin incorporation. Histograms represent the number of puromycin-positive cells (top) or the mean puromycin fluorescence intensity (bottom) relative to the mock control.

(B-C) Quantitation of GFP^+ HEK-293T cells after transduction with GFP-expressing lentiviruses. Lentivirueses expressing GFP^+ were produced in FAM46C- or D90G-expressing HEK-293T cells either treated or not with rapamycin and were used to infect fresh HEK-293T cells. The number of GFP^+ cells was assessed by either flow cytometry (B) or fluorescence microscopy (C).

(B) (Left) Representative zebra plots showing the percentage of GFP^+ cells. (Right) Histograms representing the means +/- SD of three independent experiments.

(C) Representative images of HEK-293T cells infected with GFP-expressing lentiviruses.

(D) Level of autophagic markers in HEK-293T cells downmodulated for ATG5. HEK-293T cells were transduced with lentiviral vectors expressing either a control construct (scramble) or an shRNA targeting ATG5 (ATG5 sh). ATG5 downmodulation was confirmed through RT-qPCR (top) and cytoplasmic lysates derived from the samples were subjected to SDS-PAGE and western blotting using α-ATG5, α-p62 and α-LC3 antibodies (bottom). GAPDH was used as loading control. Histograms represent the means +/- SD of three independent experiments.

Statistical *P*-values were calculated using double-tailed unpaired *t* tests. ns, $P > 0.05$; ****, $P < 0.0001$.

(E) GAG viral protein levels in HEK-293T cells downmodulated for ATG5 and expressing either wt FAM46C or the D90G mutant allele. The HEK-293T cell lines described in D and expressing either an empty vector, wt FAM46C or the D90G mutant allele were transfected with plasmids encoding for the lentiviral GAG/POL, VSV-G and REV genes. Samples were then lysed and subjected to SDS-PAGE and western blotting to assess intracellular Gag protein levels using α–p24 antibodies. GAPDH was used as loading control. Blots are representative of three independent experiments.

(F) GAG viral protein levels in HEK-293T cells upon BTZ administration.

HEK-293T cells expressing either an empty vector, FAM46C, or the D90G mutant variant and transfected with plasmids encoding for the lentiviral GAG/POL, VSV-G and REV genes were either left untreated or treated with 1.5μM BTZ for four hours. Samples were then collected and lysed as described in (E.) Poly-ubiquitinated protein detection was used as an assay control. Blots are representative of three independent experiments.

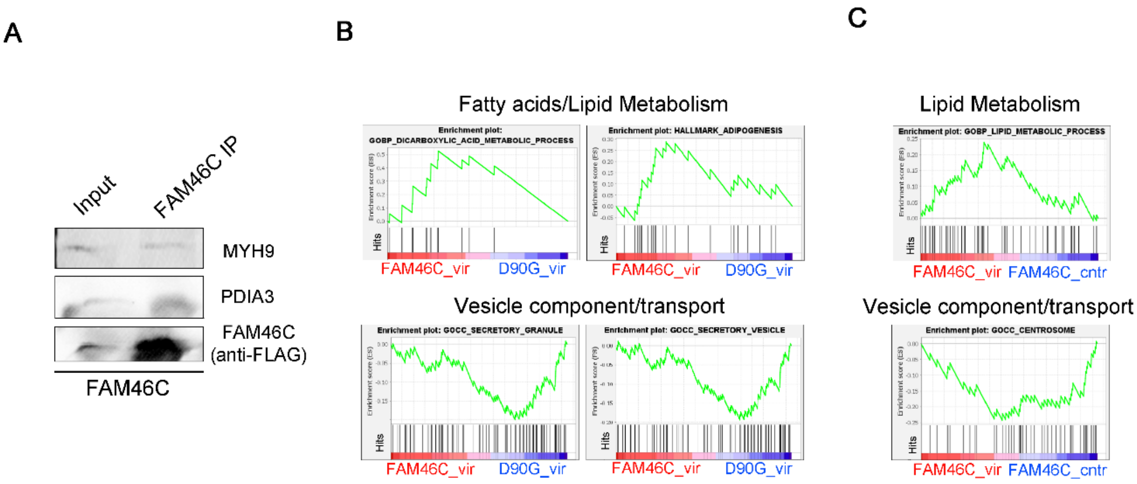

Fig.S5. The interactome of lentiviral-producing FAM46C-espressing cells is altered for proteins involved in fatty acids/lipid metabolism and vesicle transport. Related to Fig.5.

(A) Validation of FAM46C interaction with MYH9 and PDIA3. HEK-293T cells expressing wt FAM46C-FLAG were co-transfected with lentiviral envelope and packaging plasmids. FAM46C-FLAG was immunoprecipitated using anti-FLAG antibodies. Co-immunoprecipitated samples were then eluted and subjected to SDS-PAGE and western blotting to assess MYH9 and PDIA3 protein levels. A representative western blot of three independent experiments is shown.

(B-C) GSEA on the interactomes of FAM46C and D90G during lentiviral production. We analysed by GSEA the interactomes of FAM46C and D90G during viral particle production (FAM46C_vir/D90G_vir) (B) and the FAM46C interactome with its counterpart in the absence of viral production (FAM46C_vir/FAM46C_cntr) (C) highlighting fatty acids/lipid metabolism (top) and in vesicle component/transport (bottom) signatures.

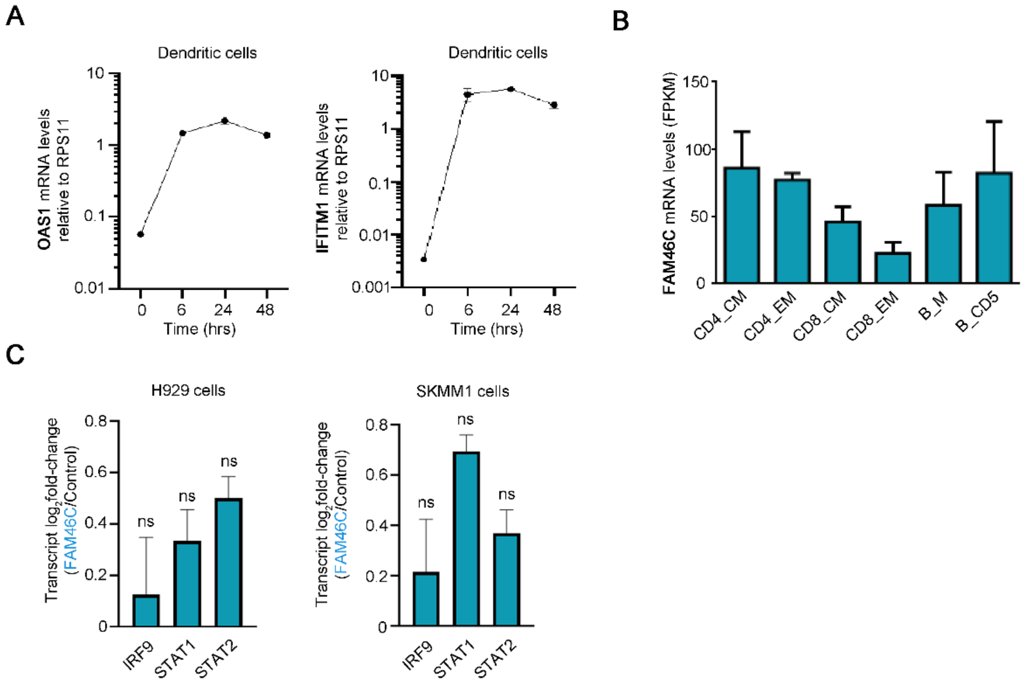

Fig.S6. mRNA level of ISGs in dendritic cells after IFN-α administration, in myeloma cells upon FAM46C overexpression and of FAM46C in different immune cell subsets. Related to Fig. 6.

(A) OAS1 and IFITM1 mRNA levels upon IFN-α administration. Dendritic cells were isolated and treated as described in Fig. 6B. Transcript levels were determined by RT-qPCR with normalization on RPS11.

(B) FAM46C mRNA levels in different immune cell subsets. Data were retrieved from (29). Histograms represent mean FPKM levels +/- SD of 4 biological replicates.

(C) ISG mRNA levels upon FAM46C overexpression in multiple myeloma cell lines. Data were retrieved from (25). Histograms represent mean $\log_2$ fold-changes +/- SE of 3 biological replicates. ns: $\log_2$ fold-change < 1 and/or *P*-value < 0.05.

Sign FAM46C +/ FAM46C-	Sign D90G+ / D90G-	*p-value* FAM46C +/ FAM46C-	Diff FAM46C +/ FAM46C-	*p-value* 090G+ / D09G-	Diff D09G+ / D90G-	Protein names	Gene names
+		1,54	3,18	0,68	0,46	Myosin-9	MYH9
+	+	1,48	2,20	1,77	2,15	FAS-associated factor 2	FAF2
+		1,78	2,20	1,23	2,20	Protein disulfide-isomerase A3	PDIA3
+		2,30	1,92	1,10	1,76	THO complex subunit 2	THOC2
+		1,34	1,75	0,01	0,01	Eukaryotic translation initiation factor 3 subunit A	EIF3A
+		1,86	1,71	0,26	-0,83	40S ribosomal protein S8	RPS8
+		2,76	1,70	0,42	0,91	Bifunctional glutamate/proline--tRNA ligase;Glutamate--tRNA ligase;Proline--tRNA ligase	EPRS
+	+	1,34	1,67	1,53	-0,79	Serine/arginine-rich splicing factor 11	SRSF11
+		1,35	1,56	0,28	-0,31	Peptidyl-prolyl cis-trans isomerase B	PPIB
+	+	1,52	1,41	1,61	2,44	Bystin	BYSL
+		4,03	1,24	0,65	0,98	WD repeat-containing protein 5	WDR5
+		1,68	1,15	0,01	-0,02	Protein PRRC2A	PRRC2A
+		1,51	1,12	0,69	-1,24	PHD finger protein 6	PHF6
+		1,45	1,09	0,48	-0,35	Aspartate aminotransferase, mitochondrial	GOT2
+	+	1,97	1,03	1,65	1,86	E3 ubiquitin-protein ligase CHIP	STUB1
+		1,74	1,00	0,19	-0,36	Cell division cycle 5-like protein	CDC5L
+		1,89	0,99	0,76	-0,42	RNA-binding protein 39	RBM39
+	+	1,52	0,93	1,98	-0,57	Purine nucleoside phosphorylase	PNP
+		3,40	0,89	1,09	1,86	SWI/SNF complex subunit SMARCC1	SMARCC1
+		1,35	0,86	0,30	-0,46	Eukaryotic translation initiation factor 3 subunit F	EIF3F
+		2,86	0,86	0,12	-0,16	Mitotic checkpoint protein BUB3	BUB3
+		1,36	0,85	0,54	1,11	Monoacylglycerol lipase ABHD12	ABHD12
+		1,63	0,81	0,39	0,41	Endoplasmic reticulum resident protein 44	ERP44
+		1,60	0,80	0,75	-1,03	U4/U6.U5 tri-snRNP-associated protein 2	USP39
+		1,62	0,80	0,78	0,86	Nucleolar protein 58	NOP58
+	+	2,08	0,78	1,83	1,07	Elongation factor Tu, mitochondrial	TUFM
+		3,82	0,75	0,79	0,44	Nuclear mitotic apparatus protein 1	NUMA1
+	+	1,46	0,72	2,10	0,36	THO complex subunit 6 homolog	THOC6
+		1,94	0,71	0,65	2,44	RuvB-like 2	RUVBL2
+		1,44	0,71	0,70	-0,82	Protein FRG1	FRG1
+		1,37	0,70	0,04	0,04	Polymerase delta-interacting protein 3	POLDIP3
+	+	1,63	0,66	1,45	-0,56	Peroxiredoxin-6	PRDX6
+		1,43	0,62	0,31	-0,11	Actin, cytoplasmic 1;Actin, cytoplasmic 1, N-terminally processed	ACTB
+	+	1,87	0,60	1,82	2,30	BCL-6 corepressor	BCOR
+		1,51	0,59	1,24	-1,76	Transaldolase	TALDO1
+		1,38	0,57	0,94	0,30	Actin-related protein 3	ACTR3
+		1,33	0,54	0,29	0,13	Nicalin	NCLN
+		1,54	0,52	0,84	-0,90	Multiple myeloma tumor-associated protein 2	MMTAG2

+		1,69	0,49	1,20	0,43	Heterogeneous nuclear ribonucleoproteins C1/C2;Heterogeneous nuclear ribonucleoprotein C-like 2;Heterogeneous nuclear ribonucleoprotein C-like 4;Heterogeneous nuclear ribonucleoprotein C-like 1;Heterogeneous nuclear ribonucleoprotein C-like 3	HNRNPC;HNRNPCL2;HNRNPCL4;HNRNPCL1;HNRNPCL3
+		2,91	0,47	0,85	-0,66	Malate dehydrogenase, mitochondrial;Malate dehydrogenase	MDH2
+		1,54	0,46	0,82	0,53	Ribose-phosphate pyrophosphokinase 1	PRPS1
+		1,62	0,43	0,31	-0,19	Annexin A5;Annexin	ANXA5
+		1,99	0,42	0,60	-0,10	DNA-dependent protein kinase catalytic subunit	PRKDC
+		2,01	0,38	0,23	0,54	Sphingosine-1-phosphate lyase 1	SGPL1
+		1,48	0,37	0,53	0,88	T-complex protein 1 subunit beta	CCT2
+		2,09	0,30	0,25	-0,21	26S proteasome non-ATPase regulatory subunit 11	PSMD11
+		2,29	-0,27	1,27	-0,84	Importin-7	IPO7
+	+	1,72	-0,30	1,49	0,99	Probable ATP-dependent RNA helicase DDX17	DDX17
+	+	1,40	-0,32	1,32	-0,77	Fructose-bisphosphate aldolase A;Fructose-bisphosphate aldolase	ALDOA
+		1,36	-0,38	1,19	0,64	DnaJ homolog subfamily A member 1	DNAJA1
+		1,41	-0,41	0,03	-0,04	Casein kinase I isoform alpha;Casein kinase I isoform alpha-like	CSNK1A1;CSNK1A1L
+		1,50	-0,46	0,86	-0,48	Mannose-1-phosphate guanyltransferase alpha	GMPPA
+		1,39	-0,48	0,00	0,00	Homeobox protein Hox-A5	HOXA5
+	+	1,60	-0,52	2,10	-0,76	L-lactate dehydrogenase A chain	LDHA
+		1,76	-0,55	0,78	0,53	2-amino-3-ketobutyrate coenzyme A ligase, mitochondrial	GCAT
+		1,44	-0,58	0,14	0,06	High mobility group protein B1;Putative high mobility group protein B1-like 1	HMGB1;HMGB1P1
+		1,32	-0,64	0,97	-0,62	Rho guanine nucleotide exchange factor 39	ARHGEF39
+		1,96	-0,64	0,09	0,13	Leucine-rich repeat and calponin homology domain-containing protein 2	LRCH2
+		2,23	-0,64	0,31	0,14	Heterogeneous nuclear ribonucleoprotein F;Heterogeneous nuclear ribonucleoprotein F, N-terminally processed	HNRNPF
+		2,81	-0,65	0,48	0,39	Desmoglein-1	DSG1
+	+	1,42	-0,67	1,32	-0,77	Acidic fibroblast growth factor intracellular-binding protein	FIBP
+		1,81	-0,68	0,85	0,47	Protein FAM46C	FAM46C
+		1,50	-0,68	0,20	0,10	Bleomycin hydrolase	BLMH
+		1,54	-0,69	0,21	-0,20	Ornithine aminotransferase, mitochondrial;Ornithine aminotransferase, hepatic form;Ornithine aminotransferase, renal form	OAT
+		1,35	-0,74	0,86	-0,76	Proliferation-associated protein 2G4	PA2G4
+		1,36	-0,74	1,03	-0,75	Tyrosyl-DNA phosphodiesterase 2	TDP2
+		1,38	-0,74	0,06	0,03	Plakophilin-2	PKP2
+	+	1,52	-0,78	1,32	1,32	Fatty acid-binding protein, epidermal	FABP5
+		1,33	-0,80	1,27	-2,18	Uncharacterized protein CXorf57	CXorf57

+		3,32	-0,81	0,15	-0,06	Pyrroline-5-carboxylate reductase;Pyrroline-5-carboxylate reductase 1, mitochondrial	PYCR1
+		2,42	-0,82	0,03	-0,06	Galactokinase	GALK1
+		1,59	-0,84	0,16	0,62	E3 ubiquitin-protein ligase TRIM21	TRIM21
+		2,23	-0,87	0,58	-0,63	Elongation factor 1-alpha 1;Putative elongation factor 1-alpha-like 3;Elongation factor 1-alpha;Elongation factor 1-alpha 2	EEF1A1;EEF1A1P5;EEF1A2
+		1,94	-0,89	0,09	-0,08	Ribosomal protein S6 kinase alpha-3	RPS6KA3
+	+	1,55	-0,93	1,44	-0,55	Unconventional myosin-Ic	MYO1C
+		1,53	-0,96	0,33	-0,63	Chromobox protein homolog 6	CBX6
+	+	1,41	-0,98	2,99	-2,13	Serine/threonine-protein kinase MARK2	MARK2
+		1,74	-1,11	0,78	0,62	Serine/threonine-protein phosphatase PP1-alpha catalytic subunit;Serine/threonine-protein phosphatase	PPP1CA
+	+	1,88	-1,15	1,53	-1,13	Elongation factor 1-delta	EEF1D
+	+	2,03	-1,15	2,41	-1,46	Cytoplasmic tRNA 2-thiolation protein 1	CTU1
+	+	1,39	-1,19	1,77	-1,14	Myristoylated alanine-rich C-kinase substrate	MARCKS
+		1,55	-1,31	1,01	-0,78	NADH-ubiquinone oxidoreductase 75 kDa subunit, mitochondrial	NDUFS1
+		1,72	-1,33	0,43	-0,70	AFG3-like protein 2	AFG3L2
+		1,74	-1,37	0,14	-0,18	A-kinase anchor protein 8-like	AKAP8L
+		1,68	-1,45	1,27	-0,37	Cell cycle and apoptosis regulator protein 2	CCAR2
+	+	1,71	-1,52	1,47	-0,87	Tropomyosin alpha-3 chain	TPM3
+		2,19	-1,72	0,30	-0,34	Puromycin-sensitive aminopeptidase;Puromycin-sensitive aminopeptidase-like protein	NPEPPS;NPEPPSL1
+	+	2,72	-1,72	4,36	-2,15	Zinc finger and BTB domain-containing protein 9	ZBTB9
+	+	1,37	-1,73	2,14	-1,65	G2 and S phase-expressed protein 1	GTSE1
+		1,93	-1,76	0,04	-0,08	14-3-3 protein eta	YWHAH
+		2,24	-1,86	0,76	-0,34	Cytohesin-1;Cytohesin-2;Cytohesin-3	CYTH2;CYTH1;CYTH3
+		1,46	-2,09	0,43	-0,74	Centrosomal protein of 85 kDa-like	CEP85L
+		3,40	-2,13	0,85	0,68	Protein PML	PML
+		1,42	-2,55	0,35	-0,66	Cell division cycle 7-related protein kinase	CDC7
	+	1,03	2,60	2,58	2,23	ATP-binding cassette sub-family D member 3	ABCD3
		1,08	2,47	1,12	-0,75	Splicing factor U2AF 65 kDa subunit	U2AF2
		0,39	2,04	0,52	-0,51	60S ribosomal protein L24	RPL24
		0,50	1,97	1,21	1,57	Ras GTPase-activating-like protein IQGAP1	IQGAP1
		0,29	1,90	0,66	-0,48	Splicing factor, proline- and glutamine-rich	SFPQ
		1,08	1,87	0,08	-0,08	General transcription factor 3C polypeptide 1	GTF3C1
	+	0,49	1,78	3,57	-1,15	Glutathione S-transferase P	GSTP1
		0,85	1,70	0,52	1,80	Calreticulin	CALR
	+	1,13	1,67	1,72	1,65	Leucine-rich PPR motif-containing protein, mitochondrial	LRPPRC
		1,17	1,65	0,99	-2,06	Luc7-like protein 3	LUC7L3
		1,12	1,63	0,73	-1,07	60S ribosomal protein L13a;Putative 60S ribosomal protein L13a protein RPL13AP3	RPL13A;RPL13a;RPL13AP3
		0,30	1,53	1,18	2,01	Catenin delta-1	CTNND1

		0,99	1,48	0,11	-0,14	3-hydroxyacyl-CoA dehydrogenase type-2	HSD17B10
	+	0,45	1,44	3,60	3,86	Heterogeneous nuclear ribonucleoprotein H2	HNRNPH2
	+	0,95	1,44	1,67	0,76	Eukaryotic translation initiation factor 4 gamma 1	EIF4G1
		1,25	1,43	0,59	0,39	Pre-mRNA-processing factor 19	PRPF19
		0,95	1,40	0,01	-0,02	Ras-related protein Rab-5C	RAB5C
		1,26	1,33	0,03	-0,05	60S ribosomal protein L11	RPL11
		0,78	1,33	0,09	0,10	Serine hydroxymethyltransferase, mitochondrial;Serine hydroxymethyltransferase	SHMT2
	+	0,64	1,31	1,37	0,96	Interferon regulatory factor 2-binding protein 1	IRF2BP1
		0,82	1,30	0,68	-1,01	Arginine and glutamate-rich protein 1	ARGLU1
	+	0,39	1,29	2,21	1,75	tRNA-splicing ligase RtcB homolog	RTCB
		0,58	1,28	0,71	-1,22	Calnexin	CANX
		0,42	1,27	0,17	0,24	Histone deacetylase 2	HDAC2
		0,73	1,24	0,48	-0,43	Prolactin-inducible protein	PIP
	+	1,10	1,23	1,35	-0,86	Polyadenylate-binding protein 4;Polyadenylate-binding protein	PABPC4
		0,84	1,23	0,94	-0,56	Heat shock protein beta-1	HSPB1
		1,06	1,23	0,17	0,23	Coiled-coil domain-containing protein 124	CCDC124
	+	0,63	1,22	2,84	-1,20	Calmodulin-regulated spectrin-associated protein 3	CAMSAP3
		0,48	1,22	0,33	-0,29	Delta(24)-sterol reductase	DHCR24
	+	1,04	1,22	1,33	1,78	RNA-binding protein 27	RBM27
		0,63	1,18	0,01	-0,03	Translational activator GCN1	GCN1L1
	+	0,27	1,17	2,35	-1,25	Rho GDP-dissociation inhibitor 1	ARHGDIA
		0,83	1,16	0,37	0,98	Lactotransferrin;Lactoferricin-H;Kaliocin-1;Lactoferroxin-A;Lactoferroxin-B;Lactoferroxin-C	LTF
		1,02	1,16	0,48	-0,27	Myosin-10	MYH10
		1,01	1,15	0,37	-0,41	Heat shock 70 kDa protein 4	HSPA4
		0,84	1,14	0,17	0,19	C-terminal-binding protein 2	CTBP2;CTBP1
	+	0,52	1,14	2,19	-0,82	Aspartate--tRNA ligase, mitochondrial	DARS2
		0,62	1,13	0,39	0,36	Golgi to ER traffic protein 4 homolog	GET4
		0,47	1,13	0,24	-0,32	Plastin-3	PLS3
		0,48	1,12	0,06	-0,04	Trifunctional enzyme subunit alpha, mitochondrial;Long-chain enoyl-CoA hydratase;Long chain 3-hydroxyacyl-CoA dehydrogenase	HADHA
		1,12	1,12	0,48	1,59	ATP synthase subunit beta, mitochondrial;ATP synthase subunit beta	ATP5B
		1,24	1,12	0,87	-0,77	Probable glutathione peroxidase 8;Glutathione peroxidase	GPX8
	+	0,44	1,11	2,44	1,25	MICOS complex subunit MIC60	IMMT
		0,90	1,10	0,91	0,78	Heat shock protein 105 kDa	HSPH1
		0,81	1,09	0,27	-0,07	Protein LSM12 homolog	LSM12
		0,85	1,09	0,69	1,34	Long-chain fatty acid transport protein 4	SLC27A4
	+	1,03	1,09	1,49	1,94	Lamin-B receptor	LBR
		1,29	1,03	0,01	0,01	Splicing factor 3A subunit 1	SF3A1
		0,40	1,02	0,00	0,00	Threonine--tRNA ligase, mitochondrial	TARS2

		0,53	1,02	0,97	1,40	RuvB-like 1	RUVBL1
		0,95	1,01	0,12	-0,14	THO complex subunit 1	THOC1
		0,48	1,01	0,69	-1,24	Microtubule-associated protein;Microtubule-associated protein 4	MAP4
		0,56	1,00	0,63	0,95	60S ribosomal protein L3	RPL3
		1,12	0,98	0,43	0,83	Inosine-5-monophosphate dehydrogenase 2	IMPDH2
		0,66	0,98	1,01	-0,31	Fibronectin type-III domain-containing protein 3A	FNDC3A
		0,56	0,96	1,04	-1,10	Poly(U)-binding-splicing factor PUF60	PUF60
	+	1,27	0,95	1,34	-1,62	Putative RNA-binding protein Luc7-like 2	LUC7L2
		0,41	0,94	0,23	-1,27	Nuclease-sensitive element-binding protein 1	YBX1
		0,49	0,94	0,54	0,88	Structural maintenance of chromosomes protein;Structural maintenance of chromosomes protein 4	SMC4
		0,69	0,93	0,13	0,08	RNA-binding protein 25	RBM25
		0,21	0,93	0,34	-0,45	T-complex protein 1 subunit eta	CCT7
		0,65	0,91	0,51	0,53	26S proteasome non-ATPase regulatory subunit 14	PSMD14
		0,43	0,90	0,00	0,00	Pre-mRNA-processing factor 40 homolog A	PRPF40A
		0,37	0,90	0,87	1,36	Spliceosome RNA helicase DDX39B	DDX39B
		1,23	0,90	0,02	0,03	Signal recognition particle receptor subunit beta	SRPRB
		0,61	0,88	0,48	0,63	Monofunctional C1-tetrahydrofolate synthase, mitochondrial	MTHFD1L
		0,71	0,87	0,64	1,36	ATP synthase subunit alpha, mitochondrial	ATP5A1
		0,69	0,87	0,17	-0,14	Small nuclear ribonucleoprotein-associated proteins B and B;Small nuclear ribonucleoprotein-associated protein N	SNRPN;SNRPB
	+	0,45	0,86	1,96	1,74	Tricarboxylate transport protein, mitochondrial	SLC25A1
		1,19	0,85	0,28	0,21	Fanconi anemia group I protein	FANCI
		0,42	0,85	0,12	0,18	Probable ubiquitin carboxyl-terminal hydrolase FAF-X	USP9X
		0,70	0,85	0,37	0,33	Heterogeneous nuclear ribonucleoprotein H3	HNRNPH3
		0,32	0,84	0,10	-0,12	Ig kappa chain C region	IGKC
	+	1,17	0,84	1,38	0,87	Regulation of nuclear pre-mRNA domain-containing protein 1A	RPRD1A
		0,63	0,84	0,07	0,06	Protein ECT2	ECT2
		0,64	0,83	0,07	0,06	Adenylate kinase 2, mitochondrial;Adenylate kinase 2, mitochondrial;Adenylate kinase 2, mitochondrial, N-terminally processed	AK2
	+	1,26	0,83	2,59	0,37	Transcription activator BRG1	SMARCA4
		0,77	0,82	0,63	-0,74	Dimethyladenosine transferase 1, mitochondrial	TFB1M
		0,70	0,81	0,17	-0,29	Peptidyl-prolyl cis-trans isomerase FKBP4;Peptidyl-prolyl cis-trans isomerase FKBP4, N-terminally processed	FKBP4
	+	0,22	0,81	1,67	-5,59	Serine/threonine-protein kinase 38	STK38
		0,59	0,80	0,78	-0,52		RPL10
	+	0,85	0,80	1,37	1,05	Vimentin	VIM
		0,85	0,80	0,63	0,42	Small ubiquitin-related modifier 2;Small ubiquitin-related modifier 4;Small ubiquitin-related modifier 3	SUMO2;SUMO3;SUMO4

		0,62	0,79	0,52	-0,76	Replication factor C subunit 1	RFC1
		0,87	0,78	0,25	-0,25	Host cell factor 1;HCF N-terminal chain 1;HCF N-terminal chain 2;HCF N-terminal chain 3;HCF N-terminal chain 4;HCF N-terminal chain 5;HCF N-terminal chain 6;HCF C-terminal chain 1;HCF C-terminal chain 2;HCF C-terminal chain 3;HCF C-terminal chain 4;HCF C-terminal chain 5;HCF C-terminal chain 6	HCFC1
		0,67	0,78	0,16	0,08	Peroxisomal multifunctional enzyme type 2;(3R)-hydroxyacyl-CoA dehydrogenase;Enoyl-CoA hydratase 2	HSD17B4
		1,05	0,77	0,72	-0,55	Pre-mRNA-splicing factor ATP-dependent RNA helicase DHX15	DHX15
		0,33	0,77	0,32	0,74	Probable ATP-dependent RNA helicase DDX6	DDX6
	+	1,24	0,76	2,38	1,04	Polypyrimidine tract-binding protein 1	PTBP1
		0,99	0,76	1,16	0,98	Monocarboxylate transporter 1	SLC16A1
		0,80	0,76	0,50	-0,25	Adenine phosphoribosyltransferase	APRT
	+	1,09	0,76	2,27	-1,19	Delta-1-pyrroline-5-carboxylate synthase;Glutamate 5-kinase;Gamma-glutamyl phosphate reductase	ALDH18A1
		0,57	0,75	0,18	0,18	SAP30-binding protein	SAP30BP
	+	0,48	0,74	2,01	1,73	Serine palmitoyltransferase 2	SPTLC2
		0,31	0,73	0,04	0,06	Elongator complex protein 1	IKBKAP
		0,60	0,72	0,68	-0,54	60S ribosomal protein L18	RPL18
	+	0,38	0,72	1,93	1,77	Aldehyde dehydrogenase X, mitochondrial	ALDH1B1
		0,40	0,71	0,20	0,14	60S ribosomal protein L27a	RPL27A
		0,22	0,71	0,04	0,02	GMP synthase [glutamine-hydrolyzing]	GMPS
		0,95	0,70	0,07	0,04	Sodium/potassium-transporting ATPase subunit alpha-1	ATP1A1
		0,44	0,69	0,32	-0,11	BUB3-interacting and GLEBS motif-containing protein ZNF207	ZNF207
		1,05	0,69	0,09	-0,13	60S ribosomal protein L17	RPL17;RPL17-C18orf32
	+	0,47	0,69	1,72	2,93	Ubiquitin-associated domain-containing protein 2	UBAC2
	+	0,43	0,68	1,59	1,14	Calcium-binding mitochondrial carrier protein Aralar1	SLC25A12
		1,27	0,68	1,23	0,89	Pre-mRNA 3-end-processing factor FIP1	FIP1L1
		0,74	0,67	0,16	0,23	Bifunctional methylenetetrahydrofolate dehydrogenase/cyclohydrolase, mitochondrial;NAD-dependent methylenetetrahydrofolate dehydrogenase;Methenyltetrahydrofolate cyclohydrolase	MTHFD2
		1,06	0,67	0,35	-0,35	UBX domain-containing protein 1	UBXN1
	+	0,86	0,67	2,09	1,22	E3 ubiquitin-protein ligase TRIM33	TRIM33
		0,53	0,66	0,35	-0,47	Lysine--tRNA ligase	KARS
		0,87	0,66	0,18	-0,17	Replication factor C subunit 5	RFC5
		0,22	0,65	0,21	0,78	Alpha-enolase	ENO1
		0,75	0,65	0,31	-0,31	Putative mitochondrial import inner membrane translocase subunit Tim23B;Mitochondrial import inner membrane translocase subunit Tim23	TIMM23B;TIMM23
		0,94	0,65	0,55	0,47	Splicing factor 45	RBM17

	+	0,18	0,64	1,63	1,34	Large proline-rich protein BAG6	BAG6;BAT3
		0,12	0,64	0,14	0,15	Baculoviral IAP repeat-containing protein 6	BIRC6
	+	0,34	0,64	2,69	-0,70	60S ribosomal protein L14	RPL14
		0,53	0,64	0,01	0,02	Eukaryotic translation initiation factor 3 subunit I	EIF3I
		0,43	0,63	0,91	-1,01	Phosphoenolpyruvate carboxykinase [GTP], mitochondrial	PCK2
		0,73	0,63	0,02	0,02	Interleukin enhancer-binding factor 3	ILF3
	+	0,83	0,61	2,23	0,96	Mitochondrial dicarboxylate carrier	SLC25A10
		0,60	0,61	0,37	-0,70	Eukaryotic translation initiation factor 2 subunit 3;Putative eukaryotic translation initiation factor 2 subunit 3-like protein	EIF2S3;EIF2S3L
		1,03	0,60	0,31	0,31	Leucine-rich repeat-containing protein 59	LRRC59
		0,49	0,60	0,39	-0,34	Serine/threonine-protein kinase A-Raf	ARAF
		0,49	0,60	0,80	1,30	La-related protein 4	LARP4
	+	0,61	0,60	1,55	-0,43	Caseinolytic peptidase B protein homolog	CLPB
		0,93	0,59	0,54	0,53	DnaJ homolog subfamily B member 1	DNAJB1
		1,22	0,59	0,45	0,24	ATPase WRNIP1	WRNIP1
		0,75	0,59	0,95	2,18	Zinc finger CCCH domain-containing protein 11A	ZC3H11A
		0,41	0,59	0,02	-0,03	NADH-cytochrome b5 reductase 3;NADH-cytochrome b5 reductase 3 membrane-bound form;NADH-cytochrome b5 reductase 3 soluble form	CYB5R3
		0,97	0,59	0,98	0,67	Structural maintenance of chromosomes protein 3	SMC3
	+	0,17	0,59	2,35	1,62	Serine/arginine repetitive matrix protein 2	SRRM2
		0,46	0,59	0,31	1,05	40S ribosomal protein S5;40S ribosomal protein S5, N-terminally processed	RPS5
	+	0,98	0,59	1,62	1,58	Heterogeneous nuclear ribonucleoprotein H;Heterogeneous nuclear ribonucleoprotein H, N-terminally processed	HNRNPH1
		0,23	0,58	0,26	-0,68	BTB/POZ domain-containing protein KCTD5	KCTD5
		1,15	0,58	1,02	0,62	Polyubiquitin-C;Ubiquitin;Ubiquitin-60S ribosomal protein L40;Ubiquitin;60S ribosomal protein L40;Ubiquitin-40S ribosomal protein S27a;Ubiquitin;40S ribosomal protein S27a;Polyubiquitin-B;Ubiquitin	UBC;UBB;RPS27A;UBA52
		0,24	0,58	0,30	-0,28	Exosome complex component RRP41	EXOSC4
		0,72	0,57	0,64	0,77	TAR DNA-binding protein 43	TARDBP;TDP43
		0,42	0,57	0,26	-0,55	Splicing factor U2AF 35 kDa subunit;Splicing factor U2AF 26 kDa subunit	U2AF1;U2AF1L4
		0,81	0,56	0,15	-0,39	Transducin beta-like protein 2	TBL2
		0,23	0,55	0,11	-0,15	Proteasome subunit beta type-4	PSMB4
		0,22	0,54	0,85	0,85	F-actin-capping protein subunit alpha-1	CAPZA1
		0,87	0,54	0,90	0,66	Very-long-chain enoyl-CoA reductase	TECR
		0,39	0,54	0,61	0,60	Choline/ethanolaminephosphotransferase 1	CEPT1
	+	0,37	0,53	3,69	-0,79	Calcineurin B homologous protein 1	CHP1
		0,64	0,52	0,11	0,06	RNA-binding protein 8A	RBM8A
	+	0,68	0,52	1,59	-1,58	Protein disulfide-isomerase A4	PDIA4

		0,29	0,51	0,33	0,27	Coatomer subunit alpha;Xenin;Proxenin	COPA
		0,81	0,51	1,06	-0,66	Stress-induced-phosphoprotein 1	STIP1
		0,54	0,50	0,21	-0,46	Ras-related protein Rab-14	RAB14
	+	0,97	0,50	1,45	-0,61	U5 small nuclear ribonucleoprotein 40 kDa protein	SNRNP40
		0,49	0,50	0,05	0,09	Nucleophosmin	NPM1
		0,62	0,50	0,15	-0,34	Casein kinase II subunit alpha	CSNK2A2
		0,80	0,49	0,43	0,38	Exportin-1	XPO1
		0,79	0,49	0,53	0,38	Electron transfer flavoprotein subunit alpha, mitochondrial	ETFA
	+	0,23	0,49	1,84	-0,90	CTP synthase 1;CTP synthase	CTPS1
		0,23	0,48	0,44	-0,31	General transcription factor 3C polypeptide 5	GTF3C5
		0,75	0,48	0,11	0,45	CDP-diacylglycerol--inositol 3-phosphatidyltransferase	CDIPT
		0,67	0,47	1,10	-0,58	GTP-binding nuclear protein Ran	RAN
		0,32	0,47	0,19	-0,17	Heat shock protein 75 kDa, mitochondrial	TRAP1
		0,82	0,46	1,15	-0,72	Clathrin heavy chain;Clathrin heavy chain 1	CLTC
		0,62	0,46	0,22	-0,30	Dolichyl-diphosphooligosaccharide--protein glycosyltransferase 48 kDa subunit	DDOST
		0,78	0,46	0,38	0,26	tRNA (cytosine(34)-C(5))-methyltransferase	NSUN2
		0,44	0,45	0,19	-0,26	Histone acetyltransferase type B catalytic subunit	HAT1
	+	0,50	0,45	2,93	-0,98	Poly [ADP-ribose] polymerase 1	PARP1
		1,16	0,44	0,77	-0,79	S-adenosylmethionine synthase isoform type-2	MAT2A
		0,64	0,44	0,09	-0,06	60S acidic ribosomal protein P0;60S acidic ribosomal protein P0-like	RPLP0;RPLP0P6
		1,16	0,44	1,23	0,48	Sideroflexin-1	SFXN1
		0,81	0,44	0,21	0,10	Spartin	SPG20
		0,39	0,44	0,35	-0,35	Probable ATP-dependent RNA helicase DDX47	DDX47
	+	0,20	0,43	3,29	1,82	Structural maintenance of chromosomes protein 1A;Structural maintenance of chromosomes protein	SMC1A
		0,85	0,42	0,75	0,61	28S ribosomal protein S27, mitochondrial	MRPS27
		0,35	0,42	0,01	-0,01	DnaJ homolog subfamily B member 4	DNAJB4
		0,16	0,42	0,34	-0,51	Regulator of nonsense transcripts 3B	UPF3B
		0,58	0,42	0,53	0,29	Valine--tRNA ligase	VARS
		0,91	0,42	0,59	-0,35	Unconventional myosin-VI	MYO6
		0,25	0,42	0,14	0,09	40S ribosomal protein S2	RPS2
		0,38	0,41	0,05	0,09	NF-kappa-B-repressing factor	NKRF
	+	1,11	0,41	1,74	0,95	ELAV-like protein 1	ELAVL1
		1,10	0,40	0,84	0,55	Lamina-associated polypeptide 2, isoform alpha;Thymopoietin;Thymopentin	TMPO
		0,27	0,40	0,79	0,60	Solute carrier family 35 member E1	SLC35E1
		0,77	0,40	0,17	0,08	Cyclin-dependent kinase 9	CDK9
		0,45	0,40	0,56	0,49	Survival motor neuron protein	SMN1;SMN2
		0,45	0,39	0,06	-0,08	Isoleucine--tRNA ligase, cytoplasmic	IARS
		0,55	0,39	0,09	-0,09	26S protease regulatory subunit 10B	PSMC6
	+	0,38	0,39	2,30	1,48	Voltage-dependent anion-selective channel protein 2	VDAC2

		0,34	0,39	1,12	-1,18	Probable ATP-dependent RNA helicase DDX46	DDX46
	+	0,55	0,39	1,34	-0,91	X-ray repair cross-complementing protein 5	XRCC5
		0,27	0,39	0,39	-0,56	Complement component 1 Q subcomponent-binding protein, mitochondrial	C1QBP
		0,26	0,39	0,07	-0,20	Ig alpha-1 chain C region;Ig alpha-2 chain C region	IGHA1;IGHA2
		0,23	0,39	1,09	-2,38	Tuftelin-interacting protein 11	TFIP11
		0,22	0,38	1,12	0,74	Peptidyl-prolyl cis-trans isomerase FKBP8	FKBP8
		0,47	0,38	0,39	0,31	Dolichyl-diphosphooligosaccharide--protein glycosyltransferase subunit STT3A	STT3A
		0,22	0,37	0,64	0,44	DBIRD complex subunit ZNF326	ZNF326
		0,79	0,37	0,66	0,58	Emerin	EMD
		0,81	0,36	0,03	0,01	Interleukin enhancer-binding factor 2	ILF2
		0,18	0,36	0,28	-0,52	Sarcoplasmic/endoplasmic reticulum calcium ATPase 2	ATP2A2
		0,70	0,35	0,66	0,32	Thymidylate synthase	TYMS
		0,50	0,35	0,97	0,60	ATP-dependent RNA helicase A	DHX9
		0,31	0,34	0,54	0,59	H/ACA ribonucleoprotein complex subunit 4	DKC1
		0,71	0,34	0,42	-0,36	BAG family molecular chaperone regulator 2	BAG2
		0,26	0,34	0,49	-0,92	Phenylalanine--tRNA ligase alpha subunit	FARSA
		0,60	0,34	0,23	0,26	Polyadenylate-binding protein 2	PABPN1
		0,58	0,34	0,01	0,02	Acetyl-CoA acetyltransferase, mitochondrial	ACAT1
		0,20	0,33	0,43	0,40	E3 ubiquitin-protein ligase UBR5	UBR5
		0,48	0,33	0,34	0,47	Annexin A2;Annexin;Putative annexin A2-like protein	ANXA2;ANXA2P2
		0,90	0,33	0,22	0,26	Heterogeneous nuclear ribonucleoprotein A3	HNRNPA3
		0,36	0,33	0,20	-0,17	Coilin	COIL
		0,74	0,32	0,17	-0,13	Prohibitin-2	PHB2
		0,51	0,32	0,18	0,07	Cytoplasmic tRNA 2-thiolation protein 2	CTU2
		0,49	0,32	0,53	-0,48	Citrate synthase, mitochondrial;Citrate synthase	CS
		0,43	0,32	0,05	0,08	Histone H1.2;Histone H1.4;Histone H1.3	HIST1H1C;HIST1H1E;HIST1H1D
		0,69	0,31	0,19	0,26	Cytochrome b-c1 complex subunit 2, mitochondrial	UQCRC2
		0,75	0,31	0,12	-0,09	Medium-chain specific acyl-CoA dehydrogenase, mitochondrial	ACADM
		0,81	0,31	0,52	-0,64	Eukaryotic translation initiation factor 3 subunit G	EIF3G
		0,27	0,31	0,18	0,25	Ancient ubiquitous protein 1	AUP1
		0,28	0,30	0,19	0,31	Protein FAM46B	FAM46B
	+	0,37	0,29	2,44	0,77	General transcription factor II-I	GTF2I
	+	0,10	0,29	1,54	-1,23	Eukaryotic translation initiation factor 3 subunit B	EIF3B
		0,50	0,29	0,07	0,07	Prohibitin	PHB
		0,63	0,29	0,65	-0,43	Developmentally-regulated GTP-binding protein 1	DRG1
		0,62	0,28	0,33	0,32	Glutaminyl-peptide cyclotransferase-like protein	QPCTL
		1,06	0,28	0,15	0,20	Serine/threonine-protein phosphatase 2A catalytic subunit alpha isoform;Serine/threonine-protein phosphatase 2A catalytic subunit beta	PPP2CA;PPP2CB

						isoform;Serine/threonine-protein phosphatase	
		0,57	0,28	0,82	0,76	Ran GTPase-activating protein 1	RANGAP1
		0,09	0,28	0,82	-0,51	NADH dehydrogenase [ubiquinone] iron-sulfur protein 3, mitochondrial	NDUFS3
	+	0,72	0,28	1,65	1,32	Acylglycerol kinase, mitochondrial	AGK
		0,12	0,28	0,02	-0,06	Protein Shroom3	SHROOM3
		0,22	0,27	0,22	-0,23	Very-long-chain 3-oxoacyl-CoA reductase	HSD17B12
		0,19	0,27	0,90	0,42	RNA polymerase II-associated factor 1 homolog	PAF1
	+	0,22	0,27	1,77	0,70	Threonylcarbamoyladenosine tRNA methylthiotransferase	CDKAL1
		0,21	0,27	0,12	-0,08	Serpin B3;Serpin B4	SERPINB3;SERPINB4
		0,46	0,27	0,63	0,63	Activating molecule in BECN1-regulated autophagy protein 1	AMBRA1
		1,01	0,27	0,44	0,48	Serine/threonine-protein phosphatase PGAM5, mitochondrial	PGAM5
		0,26	0,27	0,35	0,21	Vigilin	HDLBP
		0,32	0,26	0,36	-0,48	Calcyclin-binding protein	CACYBP
		0,14	0,26	0,53	-0,81	UDP-N-acetylglucosamine--peptide N-acetylglucosaminyltransferase 110 kDa subunit	OGT
		0,22	0,26	0,08	0,08	39S ribosomal protein L4, mitochondrial	MRPL4
		0,35	0,26	0,23	-0,28	B-cell receptor-associated protein 31	BCAP31
		0,18	0,26	0,14	0,29	Protein MCM10 homolog	MCM10
		0,20	0,26	0,33	0,25	DNA-directed RNA polymerase, mitochondrial	POLRMT
		0,33	0,26	0,04	0,03	Casein kinase I isoform epsilon;Casein kinase I isoform delta	CSNK1E;CSNK1D
		0,17	0,26	1,07	1,52	Lymphoid-specific helicase	HELLS
		0,09	0,25	1,25	-2,39	Protein arginine N-methyltransferase 5;Protein arginine N-methyltransferase 5, N-terminally processed	PRMT5
		0,49	0,25	0,19	-0,22	Histone deacetylase 1	HDAC1
		0,46	0,25	0,27	-0,49	Macrophage erythroblast attacher	MAEA
		0,58	0,25	0,34	0,33	Malectin	MLEC
		0,33	0,25	0,36	-0,16	Transitional endoplasmic reticulum ATPase	VCP
		0,19	0,25	0,02	-0,05	WD repeat-containing protein 6	WDR6
	+	0,27	0,25	1,95	-1,46	Eukaryotic translation initiation factor 2 subunit 2	EIF2S2
		0,26	0,25	0,42	-0,29	Ras-related protein Rab-7a	RAB7A
		0,34	0,24	0,04	-0,05	Synaptic vesicle membrane protein VAT-1 homolog	VAT1
	+	0,13	0,24	1,48	2,94	Exonuclease 3-5 domain-containing protein 2	EXD2
		0,58	0,24	0,52	-0,36	Poly(rC)-binding protein 1	PCBP1
	+	0,73	0,24	1,99	-1,08	40S ribosomal protein S3	RPS3
		0,30	0,23	0,94	-0,36	4F2 cell-surface antigen heavy chain	SLC3A2
		0,29	0,23	0,06	-0,05	DNA repair protein RAD51 homolog 3	RAD51C
		0,44	0,23	0,14	0,35	Probable proline--tRNA ligase, mitochondrial	PARS2
	+	1,06	0,23	1,31	0,89	RNA-binding protein Raly	RALY
		0,57	0,22	0,46	-0,56	40S ribosomal protein S3a	RPS3A
		0,08	0,22	0,67	0,37	60S ribosomal protein L9	RPL9
		0,41	0,22	0,03	0,03	Mitochondrial glutamate carrier 1	SLC25A22

		0,26	0,22	0,00	0,00	SAGA-associated factor 29 homolog	CCDC101
		0,22	0,21	0,40	-0,42	Voltage-dependent anion-selective channel protein 1	VDAC1
		0,22	0,21	0,57	0,31	Ribonuclease inhibitor	RNH1
		0,37	0,21	0,45	0,54		PDIA3
	+	0,46	0,21	1,47	-1,04	40S ribosomal protein S6	RPS6
		0,25	0,21	0,95	0,83	Dimethyladenosine transferase 2, mitochondrial	TFB2M
		0,14	0,21	0,36	-0,43	Protein FAM207A	FAM207A
		0,20	0,20	0,44	-0,24	Ig kappa chain V-II region RPMI 6410;Ig kappa chain V-II region FR;Ig kappa chain V-II region Cum	IGKV A18;IGKV2D-26;IGKV2D-29;IGKV2-40;IGKV2D-30;IGKV2D-28
		0,05	0,20	0,46	-0,52	V-type proton ATPase catalytic subunit A	ATP6V1A
		0,33	0,20	0,65	1,00	Testis-specific Y-encoded-like protein 1	TSPYL1
		0,21	0,20	0,11	-0,20	Peroxiredoxin-4	PRDX4
		0,65	0,20	1,25	0,15	ATP-dependent Clp protease ATP-binding subunit clpX-like, mitochondrial	CLPX
	+	0,29	0,19	1,31	0,64	Transcriptional repressor p66-alpha	GATAD2A
		0,36	0,19	0,98	0,58	DnaJ homolog subfamily B member 11	DNAJB11
	+	0,08	0,19	2,21	-0,92	ATP-binding cassette sub-family F member 1	ABCF1
	+	0,38	0,19	1,70	-1,30	Creatine kinase B-type	CKB
		0,12	0,19	0,09	-0,18	Alanine--tRNA ligase, cytoplasmic	AARS
		0,11	0,19	0,23	-0,13	Keratin, type I cytoskeletal 18	KRT18
		0,18	0,19	0,60	0,91	WD40 repeat-containing protein SMU1;WD40 repeat-containing protein SMU1, N-terminally processed	SMU1
		0,12	0,18	0,54	-1,04	N-alpha-acetyltransferase 10	NAA10
	+	0,40	0,18	2,96	1,31	SWI/SNF-related matrix-associated actin-dependent regulator of chromatin subfamily E member 1	SMARCE1
		0,14	0,18	0,50	-0,57	FACT complex subunit SPT16	SUPT16H
	+	0,05	0,18	1,42	1,53	Exosome complex exonuclease RRP44	DIS3
		0,84	0,18	0,25	0,23	U4/U6 small nuclear ribonucleoprotein Prp4	PRPF4
		0,16	0,18	0,25	-0,54	60S ribosomal protein L21	RPL21
		0,23	0,17	0,85	-0,49	General transcription factor IIF subunit 1	GTF2F1
		0,06	0,17	0,02	-0,01	Splicing factor 3B subunit 1	SF3B1
	+	0,23	0,17	1,43	-1,22	Cytoplasmic dynein 1 heavy chain 1	DYNC1H1
		0,28	0,17	0,45	0,20	Serine/arginine-rich splicing factor 1	SRSF1
		0,43	0,17	0,51	-0,36	Activator of 90 kDa heat shock protein ATPase homolog 1	AHSA1
		0,11	0,17	0,12	0,11	F-actin-capping protein subunit beta	CAPZB
		0,19	0,17	0,84	-0,64	C-1-tetrahydrofolate synthase, cytoplasmic;Methylenetetrahydrofolate dehydrogenase;Methenyltetrahydrofolate cyclohydrolase;Formyltetrahydrofolate synthetase;C-1-tetrahydrofolate synthase, cytoplasmic, N-terminally processed	MTHFD1
		0,07	0,17	0,11	-0,14	Arginase-1	ARG1
		0,13	0,16	0,00	-0,01	ATP synthase subunit O, mitochondrial	ATP5O

	+	0,09	0,16	2,18	-1,86	E3 SUMO-protein ligase CBX4	CBX4
		0,12	0,16	0,93	1,33	Oxygen-regulated protein 1	RP1
		0,16	0,16	1,18	-1,53	Probable dimethyladenosine transferase	DIMT1
		0,16	0,16	0,50	-0,45	60S ribosomal protein L23a	RPL23A
	+	0,17	0,16	2,09	0,77	Nuclear pore complex protein Nup155	NUP155
		0,16	0,15	1,17	-0,54	Thymidine kinase, cytosolic;Thymidine kinase	TK1
		0,13	0,15	0,62	0,77	Importin subunit alpha-1	KPNA2
		0,20	0,15	0,46	-0,28	U5 small nuclear ribonucleoprotein 200 kDa helicase	SNRNP200
		0,15	0,14	0,03	-0,03	DnaJ homolog subfamily B member 6	DNAJB6
		0,05	0,14	0,33	0,49	Far upstream element-binding protein 3	FUBP3
		0,16	0,14	0,11	0,08	Replication factor C subunit 2	RFC2
	+	0,15	0,14	1,41	0,67	Ig gamma-1 chain C region	IGHG1
		0,41	0,14	0,89	0,64	Phosphate carrier protein, mitochondrial	SLC25A3
		0,10	0,14	0,39	-0,28	Protein disulfide-isomerase A5	PDIA5
		0,38	0,13	0,61	-0,41	40S ribosomal protein S4, X isoform	RPS4X
		0,27	0,13	0,12	0,13	Prolactin regulatory element-binding protein	PREB
		0,24	0,13	0,99	-0,58	Dehydrogenase/reductase SDR family member 7B	DHRS7B
		0,06	0,13	0,19	-0,25	Eukaryotic translation initiation factor 3 subunit D	EIF3D
		0,05	0,12	0,68	1,05	Transmembrane protein 33	TMEM33
	+	0,28	0,12	3,94	-4,39	BRCA2 and CDKN1A-interacting protein	BCCIP
	+	0,33	0,12	1,65	-1,50	Eukaryotic translation initiation factor 2 subunit 1	EIF2S1
		0,34	0,12	0,21	0,08	Actin-like protein 6A	ACTL6A
		0,26	0,12	0,07	-0,04	40S ribosomal protein SA	RPSA
		0,31	0,12	0,90	-0,63	DNA-directed RNA polymerase;DNA-directed RNA polymerase I subunit RPA1	POLR1A
		0,07	0,12	0,54	1,32	Drebrin	DBN1
		0,07	0,12	0,27	-0,33	Phosphatidylserine synthase 1	PTDSS1
		0,26	0,11	0,44	0,38	U1 small nuclear ribonucleoprotein 70 kDa	SNRNP70
		0,05	0,11	0,14	0,15	Serine/threonine-protein phosphatase PP1-beta catalytic subunit;Serine/threonine-protein phosphatase	PPP1CB
		0,11	0,11	0,78	-0,64		
		0,10	0,11	0,51	0,43	Endoplasmic reticulum-Golgi intermediate compartment protein 1	ERGIC1
		0,24	0,11	0,82	0,70	Fatty acyl-CoA reductase 1	FAR1
	+	0,14	0,11	1,56	0,96	Protein SON	SON
		0,14	0,10	0,41	-0,22	Insulin-like growth factor 2 mRNA-binding protein 1	IGF2BP1
		0,05	0,10	0,24	0,15	Coatomer subunit beta	COPB2
		0,07	0,10	0,94	-0,49	Neutral amino acid transporter B(0);Amino acid transporter	SLC1A5
	+	0,73	0,10	2,35	0,38	Cyclin-dependent kinase 1	CDK1;CDC2
		0,14	0,10	0,14	0,13	Cleavage and polyadenylation specificity factor subunit 1	CPSF1
		0,09	0,10	0,19	0,22	5-3 exoribonuclease 2	XRN2
		0,08	0,10	0,36	-0,20	Enoyl-CoA hydratase, mitochondrial	ECHS1

		0,13	0,10	0,11	0,15	Peroxiredoxin-1	PRDX1
		0,20	0,10	1,24	-1,03	60S ribosomal protein L4	RPL4
		0,26	0,10	0,11	0,23	Voltage-dependent anion-selective channel protein 3	VDAC3
	+	0,07	0,09	1,95	1,22	Transcription elongation regulator 1	TCERG1
		0,16	0,09	0,41	-0,21	Mitochondrial import inner membrane translocase subunit TIM44	TIMM44
		0,19	0,09	0,44	-0,50	U2 small nuclear ribonucleoprotein A	SNRPA1
	+	0,11	0,09	2,26	-0,64	Casein kinase II subunit alpha;Casein kinase II subunit alpha 3	CSNK2A1;CSNK2A3
		0,20	0,09	0,17	0,17	Eukaryotic initiation factor 4A-I	EIF4A1
		0,09	0,09	0,76	-0,69	Transcription elongation factor B polypeptide 3	TCEB3
		0,05	0,08	0,82	0,44	Dynactin subunit 1	DCTN1
	+	0,28	0,08	1,40	0,98	ATP synthase subunit gamma, mitochondrial	ATP5C1
		0,11	0,08	0,25	-0,22	Eukaryotic initiation factor 4A-III;Eukaryotic initiation factor 4A-III, N-terminally processed	EIF4A3
		0,06	0,08	0,60	-1,77	Zinc-alpha-2-glycoprotein	AZGP1
	+	0,05	0,08	1,31	1,61	HLA class I histocompatibility antigen, Cw-7 alpha chain	HLA-C;HLA-B
		0,07	0,08	0,03	0,10	Basigin	BSG
		0,04	0,08	0,75	0,76	Complex I assembly factor TIMMDC1, mitochondrial	TIMMDC1
		0,08	0,07	0,43	0,24	Sideroflexin-2	SFXN2
		0,20	0,07	1,21	-0,67	Mitochondrial 2-oxoglutarate/malate carrier protein	SLC25A11
		0,16	0,07	0,91	-0,55	Guanine nucleotide-binding protein subunit beta-2-like 1;Guanine nucleotide-binding protein subunit beta-2-like 1, N-terminally processed	GNB2L1
		0,09	0,06	1,09	-1,14	Serine/threonine-protein phosphatase;Serine/threonine-protein phosphatase PP1-gamma catalytic subunit	PPP1CC
		0,06	0,06	0,09	-0,16	40S ribosomal protein S7	RPS7
		0,03	0,06	0,60	2,04	D-3-phosphoglycerate dehydrogenase	PHGDH
		0,06	0,06	0,09	0,25	60S ribosomal protein L7a	RPL7A
		0,03	0,05	0,12	0,13	Fibronectin type III domain-containing protein 3B	FNDC3B
	+	0,03	0,05	1,44	0,96	DNA replication licensing factor MCM5;DNA helicase	MCM5
		0,04	0,05	0,59	-0,31	Cat eye syndrome critical region protein 5	CECR5
		0,14	0,05	0,65	0,29	PERQ amino acid-rich with GYF domain-containing protein 2	GIGYF2
		0,05	0,04	0,15	0,23	Rab3 GTPase-activating protein catalytic subunit	RAB3GAP1
		0,04	0,04	1,25	-0,78	Pre-mRNA-processing-splicing factor 8	PRPF8
	+	0,03	0,04	3,10	-3,66	Kinesin-like protein KIF11	KIF11
		0,03	0,04	0,65	-0,45	CDK5 regulatory subunit-associated protein 1	CDK5RAP1
		0,05	0,03	0,37	0,36	Serine/threonine-protein kinase PLK1	PLK1
	+	0,02	0,03	1,79	1,56	KH domain-containing, RNA-binding, signal transduction-associated protein 1	KHDRBS1
		0,01	0,03	0,59	1,19	Putative RNA-binding protein 15	RBM15
		0,04	0,03	0,02	-0,02	DNA-directed RNA polymerase;DNA-directed RNA polymerase II subunit RPB2	POLR2B
		0,09	0,03	0,65	1,98	Tubulin beta-6 chain	TUBB6

	+	0,05	0,03	3,69	1,17	Angiomotin	AMOT
		0,09	0,03	0,11	0,10	Serine-threonine kinase receptor-associated protein	STRAP
		0,14	0,02	0,14	0,17	Box C/D snoRNA protein 1	ZNHIT6
		0,04	0,02	1,25	0,68	Ras-related protein Rab-1B;Ras-related protein Rab-1A	RAB1B;RAB1A
		0,03	0,02	0,14	-0,14	Glutamate dehydrogenase 1, mitochondrial;Glutamate dehydrogenase 2, mitochondrial	GLUD1;GLUD2
		0,02	0,02	0,13	-0,22	60S ribosomal protein L7	RPL7
		0,01	0,02	0,10	-0,09	Protein LYRIC	MTDH
		0,05	0,02	0,47	0,35	ADP/ATP translocase 2;ADP/ATP translocase 2, N-terminally processed	SLC25A5
	+	0,01	0,02	1,94	1,30	E3 SUMO-protein ligase RanBP2	RANBP2
		0,02	0,01	0,18	0,18	Sideroflexin-4	SFXN4
		0,01	0,01	0,54	1,96	Lipocalin-1	LCN1
		0,00	0,01	0,00	0,00	Pre-mRNA-splicing factor SLU7	SLU7
		0,01	0,01	0,34	0,30	Protein-glutamine gamma-glutamyltransferase E;Protein-glutamine gamma-glutamyltransferase E 50 kDa catalytic chain;Protein-glutamine gamma-glutamyltransferase E 27 kDa non-catalytic chain	TGM3
		0,01	0,01	0,50	1,11	Zinc finger protein 318	ZNF318
		0,01	0,01	0,90	-0,26	Ribosomal protein L15;60S ribosomal protein L15	RPL15
		0,01	0,00	1,03	-0,45	Replication factor C subunit 3	RFC3
		0,01	0,00	1,19	0,41	DNA replication licensing factor MCM7	MCM7
		0,00	0,00	0,16	-0,23	Splicing factor 3B subunit 3	SF3B3
	+	0,00	0,00	1,53	-1,17	Kinesin-like protein KIF2A	KIF2A
		0,00	0,00	0,26	-0,23	MAP7 domain-containing protein 1	MAP7D1
		0,00	0,00	0,19	-0,24	Vesicle-trafficking protein SEC22b	SEC22B
		0,00	0,00	0,29	0,47	L-xylulose reductase	DCXR
		0,02	-0,01	0,96	0,52	SAFB-like transcription modulator	SLTM
	+	0,02	-0,01	1,88	1,21	Centrosomal protein of 170 kDa	CEP170
		0,01	-0,01	0,39	-0,29	Vesicle-associated membrane protein-associated protein A	VAPA
		0,06	-0,01	0,35	-0,60	Exosome complex component RRP4	EXOSC2
		0,06	-0,01	0,37	-0,24	Tubulin gamma-1 chain;Tubulin gamma-2 chain	TUBG1;TUBG2
		0,02	-0,02	1,19	-0,32	T-complex protein 1 subunit gamma	CCT3
	+	0,01	-0,03	1,68	0,47	Heterogeneous nuclear ribonucleoprotein A0	HNRNPA0
		0,09	-0,03	0,27	-0,39	60S ribosomal protein L5	RPL5
		0,03	-0,03	0,58	0,52	Nitric oxide synthase-interacting protein	NOSIP
		0,01	-0,03	0,18	-0,49	Casein kinase II subunit beta	CSNK2B;CSNK2B-LY6G5B-1181;CSNK2B-LY6G5B--991
		0,03	-0,04	0,10	-0,17	E3 ubiquitin-protein ligase MYCBP2	MYCBP2
		0,04	-0,04	0,50	0,13	Hypoxanthine-guanine phosphoribosyltransferase	HPRT1
		0,12	-0,05	0,85	2,09	Ig gamma-2 chain C region;Ig gamma-4 chain C region	IGHG2;IGHG4
	+	0,02	-0,05	2,06	1,09	Heterogeneous nuclear ribonucleoprotein U	HNRNPU
		0,02	-0,05	0,31	0,23	Myb-binding protein 1A	MYBBP1A

		0,09	-0,05	0,58	-0,16	Actin-related protein 2	ACTR2
		0,20	-0,06	0,52	-0,19	26S protease regulatory subunit 8	PSMC5
		0,06	-0,06	0,35	-0,16	Putative tRNA (cytidine(32)/guanosine(34)-2-O)-methyltransferase	FTSJ1
		0,08	-0,06	0,16	-0,13	DnaJ homolog subfamily A member 3, mitochondrial	DNAJA3
	+	0,10	-0,06	1,48	-0,76	Polymerase delta-interacting protein 2	POLDIP2
		0,08	-0,06	1,08	-0,51	Insulin receptor substrate 4	IRS4
		0,03	-0,06	0,05	0,07	Protein disulfide-isomerase	P4HB
		0,10	-0,06	0,81	-0,76	Phosphoserine aminotransferase	PSAT1
	+	0,05	-0,07	1,33	-2,45	Tyrosine-protein kinase JAK1	JAK1
		0,07	-0,07	0,63	-0,57	Cathepsin D;Cathepsin D light chain;Cathepsin D heavy chain	CTSD
		0,08	-0,07	0,09	0,09	Pyruvate dehydrogenase E1 component subunit beta, mitochondrial	PDHB
		0,13	-0,08	0,08	-0,04	Replication factor C subunit 4	RFC4
		0,03	-0,08	0,39	0,45	Metastasis-associated protein MTA2	MTA2
		0,05	-0,08	0,91	-0,73	T-complex protein 1 subunit epsilon	CCT5
		0,11	-0,08	1,25	-0,61	28S ribosomal protein S22, mitochondrial	MRPS22
	+	0,15	-0,09	1,52	-0,63	Proteasome subunit alpha type;Proteasome subunit alpha type-4;Proteasome subunit beta type	PSMA4
	+	0,17	-0,09	1,78	-0,89	Glyceraldehyde-3-phosphate dehydrogenase	GAPDH
		0,15	-0,09	0,11	-0,12	Nascent polypeptide-associated complex subunit alpha;Nascent polypeptide-associated complex subunit alpha, muscle-specific form	NACA
		0,23	-0,09	0,35	0,29	Urotensin-2	UTS2
		0,04	-0,10	0,91	1,20	Structural maintenance of chromosomes protein 2	SMC2
		0,05	-0,10	0,74	-1,61	E3 ubiquitin-protein ligase RING2	RNF2
		0,08	-0,10	0,75	1,09	NADH dehydrogenase [ubiquinone] 1 alpha subcomplex subunit 10, mitochondrial	NDUFA10
		0,20	-0,10	0,14	0,17	Sodium/potassium-transporting ATPase subunit beta-3	ATP1B3
		0,08	-0,11	0,06	0,07	Mitogen-activated protein kinase 1	MAPK1
		0,07	-0,11	0,26	-0,39	MARCKS-related protein	MARCKSL1
		0,22	-0,11	0,64	0,45	Flap endonuclease 1	FEN1
		0,11	-0,11	0,02	-0,07	Lysozyme C;Lysozyme	LYZ
		0,16	-0,11	0,02	0,05	Crk-like protein	CRKL
		0,14	-0,11	0,04	-0,04	Arginine--tRNA ligase, cytoplasmic	RARS
		0,03	-0,11	0,20	-0,26	Endoplasmin	HSP90B1
		1,11	-0,11	0,03	0,05	Spermidine synthase	SRM
		0,31	-0,11	1,03	1,05	Transcription initiation factor IIB	GTF2B
		0,08	-0,12	0,67	-0,26	YTH domain-containing family protein 2	YTHDF2
		0,44	-0,12	1,28	0,85	NADH dehydrogenase [ubiquinone] iron-sulfur protein 2, mitochondrial	NDUFS2
		0,52	-0,12	1,28	0,42	Poly(rC)-binding protein 2	PCBP2
		0,34	-0,13	0,50	-0,63	Eukaryotic translation initiation factor 3 subunit E	EIF3E
		0,22	-0,14	0,44	0,81	ATP synthase subunit s-like protein	ATP5SL
		0,19	-0,14	0,07	0,12	E3 ubiquitin-protein ligase RNF126	RNF126

		0,03	-0,14	0,22	-0,09	Staphylococcal nuclease domain-containing protein 1	SND1
	+	0,14	-0,14	1,74	-0,42	DNA polymerase delta subunit 3	POLD3
		0,09	-0,14	0,03	-0,04	Thioredoxin-dependent peroxide reductase, mitochondrial	PRDX3
	+	0,15	-0,14	1,41	-1,25	Eukaryotic translation initiation factor 5B	EIF5B
		1,15	-0,14	0,18	0,21	RNA-binding motif protein, X chromosome;RNA-binding motif protein, X chromosome, N-terminally processed;RNA binding motif protein, X-linked-like-1	RBMX;RBMXL1
	+	0,15	-0,15	2,10	-1,71	Transketolase	TKT
		0,78	-0,15	0,05	-0,03	Pyruvate dehydrogenase E1 component subunit alpha, somatic form, mitochondrial	PDHA1
		0,53	-0,15	0,29	-0,31	Heterogeneous nuclear ribonucleoprotein A/B	HNRNPAB
		0,07	-0,15	0,72	1,64	Plasminogen activator inhibitor 1 RNA-binding protein	SERBP1
		0,04	-0,15	0,62	-0,69	Cytosolic Fe-S cluster assembly factor NUBP2	NUBP2
		0,55	-0,15	0,75	-0,79	Inorganic pyrophosphatase	PPA1
		0,05	-0,15	0,47	1,06	Double-stranded RNA-specific adenosine deaminase	ADAR
	+	0,09	-0,15	1,31	-0,58	Melanoma-associated antigen D2	MAGED2
		0,14	-0,16	0,66	-0,66	Acyl-CoA desaturase	SCD
		0,07	-0,16	0,43	0,69	Pleiotropic regulator 1	PLRG1
		0,07	-0,16	0,24	0,52	Tubulin alpha-1B chain	TUBA1B
		0,43	-0,17	0,04	0,04	DnaJ homolog subfamily C member 10	DNAJC10
		0,23	-0,17	0,90	0,93	Nuclear envelope pore membrane protein POM 121C	POM121C
		0,10	-0,17	0,63	0,22	Gem-associated protein 4	GEMIN4
	+	0,28	-0,17	1,51	-0,50	Pleckstrin homology domain-containing family H member 3	PLEKHH3
		0,54	-0,18	0,79	-0,82	14-3-3 protein epsilon	YWHAE
	+	0,26	-0,18	1,34	1,10	Uridine 5-monophosphate synthase;Orotate phosphoribosyltransferase;Orotidine 5-phosphate decarboxylase	UMPS
		0,31	-0,18	0,29	0,20	Ribosomal RNA-processing protein 7 homolog A	RRP7A
	+	0,31	-0,19	2,12	-1,21	DNA-directed RNA polymerase I subunit RPA2	POLR1B
		0,09	-0,19	0,21	-0,31	Ras-related protein Rab-21	RAB21
		0,16	-0,19	0,21	0,13	U2 snRNP-associated SURP motif-containing protein	U2SURP
		0,15	-0,19	0,15	0,18	FACT complex subunit SSRP1	SSRP1
		0,70	-0,19	0,80	-0,55	Activator of basal transcription 1	ABT1
		0,16	-0,20	0,78	1,46	26S protease regulatory subunit 6B	PSMC4
	+	0,42	-0,20	1,71	1,20	HIG1 domain family member 1A, mitochondrial	HIGD1A
		0,21	-0,20	0,53	0,43	Sentrin-specific protease 1	SENP1
		0,06	-0,21	0,34	-0,42	Filamin-A	FLNA
	+	0,32	-0,21	1,35	0,56	ADP/ATP translocase 3;ADP/ATP translocase 3, N-terminally processed	SLC25A6
		0,06	-0,21	0,58	-1,17	Neutral alpha-glucosidase AB	GANAB
		0,05	-0,22	0,12	-0,08	116 kDa U5 small nuclear ribonucleoprotein component	EFTUD2
		0,28	-0,22	0,15	0,24	DNA-directed RNA polymerase I subunit RPA49	POLR1E

		0,81	-0,22	0,68	-0,75	Succinyl-CoA ligase [ADP-forming] subunit beta, mitochondrial	SUCLA2
		0,58	-0,23	0,63	-0,89	Eukaryotic translation initiation factor 5	EIF5
		0,26	-0,23	0,00	0,01	Dual specificity protein kinase TTK	TTK
		0,72	-0,23	0,26	-0,15	DnaJ homolog subfamily A member 2	DNAJA2
		0,09	-0,24	0,84	-0,75	C2 domain-containing protein 2-like	C2CD2L
		0,16	-0,24	0,18	0,82	E3 ubiquitin-protein ligase RING1	RING1
		0,25	-0,24	0,27	-0,30	cAMP-dependent protein kinase type I-alpha regulatory subunit;cAMP-dependent protein kinase type I-alpha regulatory subunit, N-terminally processed	PRKAR1A
		0,39	-0,24	1,17	1,51	Vacuolar protein sorting-associated protein 35	VPS35
	+	0,45	-0,24	1,37	0,46	ATP-dependent zinc metalloprotease YME1L1	YME1L1
		0,35	-0,25	0,05	0,03	DnaJ homolog subfamily C member 7	DNAJC7
		0,29	-0,25	0,02	-0,01	Very-long-chain (3R)-3-hydroxyacyl-CoA dehydratase 3	HACD3
		0,59	-0,25	0,42	0,50	DnaJ homolog subfamily B member 12	DNAJB12
		0,84	-0,25	0,01	0,02	Tropomodulin-3	TMOD3
		0,07	-0,25	0,48	-0,60	Proteasome subunit alpha type-7	PSMA7
		0,32	-0,25	0,48	0,43	U2 small nuclear ribonucleoprotein B	SNRPB2
		0,21	-0,26	0,69	-0,25	Protein AAR2 homolog	AAR2
		0,44	-0,26	0,54	0,34	39S ribosomal protein L37, mitochondrial	MRPL37
		0,70	-0,26	0,65	-0,72	Malate dehydrogenase, cytoplasmic;Malate dehydrogenase	MDH1
		0,13	-0,26	0,90	0,49	Insulin-like growth factor 2 mRNA-binding protein 3	IGF2BP3
		0,58	-0,26	0,49	1,76	FYVE, RhoGEF and PH domain-containing protein 4	FGD4
		1,20	-0,27	0,06	-0,05	Guanine nucleotide-binding protein G(s) subunit alpha isoforms XLas;Guanine nucleotide-binding protein G(s) subunit alpha isoforms short	GNAS
		0,34	-0,27	0,02	-0,01	Heterogeneous nuclear ribonucleoproteins A2/B1	HNRNPA2B1
		0,17	-0,27	0,06	0,05	Cullin-associated NEDD8-dissociated protein 1	CAND1
		0,50	-0,28	0,32	0,15	DNA mismatch repair protein Msh6	MSH6
		0,44	-0,28	0,21	0,29	Transmembrane protein 201	TMEM201
		0,29	-0,28	0,38	0,23	HCLS1-associated protein X-1	HAX1
		0,29	-0,28	0,39	0,59	Homeobox protein Hox-B9	HOXB9
	+	0,56	-0,29	1,49	-0,78	Heat shock protein HSP 90-alpha	HSP90AA1
		0,31	-0,29	0,52	0,18	Peroxisomal 2,4-dienoyl-CoA reductase	DECR2
		0,36	-0,29	0,46	0,69	26S proteasome non-ATPase regulatory subunit 8	PSMD8
	+	0,51	-0,29	1,47	-1,04	Phosphoglycerate kinase 1	PGK1
	+	0,16	-0,30	1,46	-1,14	Alsin	ALS2
		0,18	-0,30	0,53	-0,27	Fatty acid synthase;[Acyl-carrier-protein] S-acetyltransferase;[Acyl-carrier-protein] S-malonyltransferase;3-oxoacyl-[acyl-carrier-protein] synthase;3-oxoacyl-[acyl-carrier-protein] reductase;3-hydroxyacyl-[acyl-carrier-protein] dehydratase;Enoyl-[acyl-carrier-protein] reductase;Oleoyl-[acyl-carrier-protein] hydrolase	FASN

		0,71	-0,30	0,18	0,06	Importin subunit beta-1	KPNB1
	+	0,05	-0,31	2,22	-1,59	Pyruvate kinase PKM;Pyruvate kinase	PKM
		0,44	-0,31	0,51	-0,65	Transcription intermediary factor 1-beta	TRIM28
		1,01	-0,31	0,30	-0,19	AH receptor-interacting protein	AIP
	+	0,21	-0,31	2,70	0,75	Matrin-3	MATR3
		0,55	-0,32	0,08	0,06	Serpin H1	SERPINH1
	+	0,72	-0,32	3,46	1,07	La-related protein 4B	LARP4B
		0,37	-0,32	0,77	-0,66	Mannose-1-phosphate guanyltransferase beta	GMPPB
		0,65	-0,32	0,93	-0,66	DNA-directed RNA polymerases I and III subunit RPAC1	POLR1C
		0,36	-0,33	0,32	-0,35	DNA-directed RNA polymerases I, II, and III subunit RPABC1	POLR2E
		0,34	-0,33	1,09	0,44	RNA-binding protein 4	RBM4
		0,19	-0,33	0,07	-0,21	Pachytene checkpoint protein 2 homolog	TRIP13
		0,66	-0,35	0,88	-0,85	Cytoskeleton-associated protein 2	CKAP2
		0,26	-0,35	0,54	-0,24	26S proteasome non-ATPase regulatory subunit 2	PSMD2
	+	0,76	-0,35	1,34	-1,18	L-lactate dehydrogenase B chain;L-lactate dehydrogenase	LDHB
		0,70	-0,36	1,10	-3,25	PDZ domain-containing protein GIPC1	GIPC1
		0,61	-0,36	0,04	0,04	Transcriptional adapter 1	TADA1
		0,56	-0,36	0,26	0,38	Calponin-3;Calponin	CNN3
	+	0,26	-0,36	1,45	-1,68	Multifunctional protein ADE2;Phosphoribosylaminoimidazole-succinocarboxamide synthase;Phosphoribosylaminoimidazole carboxylase	PAICS
		0,20	-0,37	1,13	-0,92	Chromatin target of PRMT1 protein	CHTOP
		0,64	-0,38	0,95	0,98	Nuclear pore complex protein Nup205	NUP205
		0,50	-0,38	0,34	-0,39	Deoxynucleoside triphosphate triphosphohydrolase SAMHD1	SAMHD1
		0,21	-0,39	0,07	-0,20	Protein S100-A7;Protein S100-A7A	S100A7;S100A7A
		0,90	-0,39	0,08	0,19	Translocon-associated protein subunit alpha	SSR1
		0,18	-0,39	0,72	1,39	Serine palmitoyltransferase 1	SPTLC1
		0,61	-0,39	0,28	0,52	14-3-3 protein theta	YWHAQ
		0,72	-0,40	0,46	0,68	Succinate dehydrogenase [ubiquinone] flavoprotein subunit, mitochondrial	SDHA
		0,87	-0,40	0,62	-0,21	Apoptosis-inducing factor 1, mitochondrial	AIFM1
		0,55	-0,40	0,08	-0,14	Golgi resident protein GCP60	ACBD3
		0,09	-0,40	0,09	0,10	X-ray repair cross-complementing protein 6	XRCC6
		0,76	-0,40	0,55	-0,29	Mitochondrial import inner membrane translocase subunit TIM50	TIMM50
		0,32	-0,41	0,04	0,05	Dermcidin;Survival-promoting peptide;DCD-1	DCD
	+	0,74	-0,41	1,31	-0,46	Protein-L-isoaspartate O-methyltransferase;Protein-L-isoaspartate(D-aspartate) O-methyltransferase	PCMT1
		1,10	-0,41	0,50	0,37	GTPase Era, mitochondrial	ERAL1
		0,63	-0,42	1,10	-0,52	DNA topoisomerase 1	TOP1
		0,57	-0,42	0,03	0,01	Nucleus accumbens-associated protein 1	NACC1
		1,29	-0,42	0,28	0,30	Trypsin-3	PRSS3

		0,47	-0,43	1,00	-0,96	Mitotic spindle assembly checkpoint protein MAD2A	MAD2L1
		0,12	-0,44	0,63	-0,51	Dolichyl-diphosphooligosaccharide--protein glycosyltransferase subunit 1	RPN1
		0,13	-0,44	0,25	0,39	Ubiquitin carboxyl-terminal hydrolase;Ubiquitin carboxyl-terminal hydrolase 11	USP11
		0,30	-0,44	0,73	-0,56	Lamin-B1	LMNB1
		0,80	-0,44	0,18	-0,19	Sulfhydryl oxidase;FAD-linked sulfhydryl oxidase ALR	GFER
		0,23	-0,44	0,86	-0,88	60S ribosomal protein L23	RPL23
		1,12	-0,45	0,14	0,09	Nucleolin	NCL
		0,36	-0,47	0,25	0,21	ATPase family AAA domain-containing protein 3B	ATAD3B
	+	1,13	-0,48	2,00	-1,02	Protein SET;Protein SETSIP	SET;SETSIP
		0,54	-0,48	0,43	0,44	Uridine-cytidine kinase 2	UCK2
	+	0,29	-0,48	3,29	-0,81	T-complex protein 1 subunit alpha	TCP1
	+	0,73	-0,48	1,40	-1,49	Tyrosine-protein phosphatase non-receptor type 13	PTPN13
		0,45	-0,49	0,82	0,92	DnaJ homolog subfamily B member 14	DNAJB14
		0,54	-0,49	0,32	-0,38	Cytoskeleton-associated protein 5	CKAP5
		0,23	-0,49	0,30	0,35	28S ribosomal protein S7, mitochondrial	MRPS7
		0,27	-0,50	0,55	-0,44	FRAS1-related extracellular matrix protein 2	FREM2
		0,36	-0,51	0,79	0,28	S1 RNA-binding domain-containing protein 1	SRBD1
		0,27	-0,51	0,50	-1,17	ATP-citrate synthase	ACLY
		0,38	-0,51	0,40	1,53	Peroxiredoxin-2	PRDX2
	+	0,79	-0,51	2,19	-0,97	60S ribosomal protein L13	RPL13
		0,33	-0,52	0,17	-0,11	rRNA 2-O-methyltransferase fibrillarin	FBL
		0,57	-0,52	0,34	-0,29	Proteasome subunit alpha type-3	PSMA3
		1,25	-0,54	0,06	-0,06	Glutaryl-CoA dehydrogenase, mitochondrial	GCDH
		0,22	-0,54	0,86	0,29	Desmoplakin	DSP
		1,08	-0,54	0,23	-0,10	Rho GTPase-activating protein 21	ARHGAP21
	+	0,93	-0,55	1,61	0,76	DNA-3-methyladenine glycosylase	MPG
		1,18	-0,56	1,26	0,99	Protein S100-A8;Protein S100-A8, N-terminally processed	S100A8
	+	1,03	-0,57	1,63	-1,01	CAD protein;Glutamine-dependent carbamoyl-phosphate synthase;Aspartate carbamoyltransferase;Dihydroorotase	CAD
		0,98	-0,57	0,15	0,23	DDB1- and CUL4-associated factor 7	DCAF7
	+	1,04	-0,57	2,18	-0,71	Proliferating cell nuclear antigen	PCNA
		0,39	-0,58	0,00	0,02	Ribosomal protein L19;60S ribosomal protein L19	RPL19
		0,41	-0,58	0,03	0,06	Membrane-associated progesterone receptor component 1	PGRMC1
		0,28	-0,58	0,11	0,28	Tubulin beta chain	TUBB
		0,17	-0,60	0,48	-1,24	60S ribosomal protein L18a	RPL18A
		0,76	-0,60	1,13	-0,80	Crooked neck-like protein 1	CRNKL1
		0,33	-0,61	0,03	0,03	tRNA (uracil-5-)-methyltransferase homolog A	TRMT2A
	+	0,61	-0,61	1,38	-0,84	Kinesin-like protein KIF18B	KIF18B
		1,05	-0,62	0,33	0,76	Tubulin beta-4B chain	TUBB4B
		0,12	-0,62	0,59	0,45	Polyadenylate-binding protein 1;Polyadenylate-binding protein	PABPC1

		0,71	-0,65	0,38	0,85	NADH dehydrogenase [ubiquinone] 1 alpha subcomplex subunit 9, mitochondrial	NDUFA9
		0,21	-0,66	0,51	-0,40	Cancer-related nucleoside-triphosphatase	NTPCR
		1,13	-0,67	0,35	0,09	Junction plakoglobin	JUP
		0,65	-0,67	0,02	-0,01	Heterogeneous nuclear ribonucleoprotein A1;Heterogeneous nuclear ribonucleoprotein A1, N-terminally processed;Heterogeneous nuclear ribonucleoprotein A1-like 2	HNRNPA1;HNRNPA1L2
		0,42	-0,68	0,35	-0,31	Guanine nucleotide-binding protein-like 3-like protein	GNL3L
	+	0,47	-0,69	3,01	1,06	Phosphatidylinositol 4-phosphate 5-kinase type-1 alpha	PIP5K1A
		1,00	-0,70	0,24	0,31	Gamma-glutamylcyclotransferase	GGCT
		0,47	-0,70	1,18	-0,36	T-complex protein 1 subunit delta	CCT4
		0,27	-0,70	0,35	0,49	7-dehydrocholesterol reductase	DHCR7
		1,26	-0,72	0,61	-0,52	Treacle protein	TCOF1
		0,44	-0,72	0,03	-0,04	Cell division control protein 6 homolog	CDC6
		0,96	-0,74	0,10	0,21	Nuclear distribution protein nudE homolog 1	NDE1
		0,47	-0,75	0,53	0,31	Dedicator of cytokinesis protein 7	DOCK7
		0,44	-0,75	0,30	-0,38	Aurora kinase A	AURKA
		0,39	-0,76	0,20	0,38	Tubulin beta-2A chain;Tubulin beta-2B chain	TUBB2A;TUBB2B
		1,00	-0,77	0,31	-0,48	Serine/threonine-protein phosphatase 2A 65 kDa regulatory subunit A alpha isoform	PPP2R1A
		0,47	-0,79	0,17	0,48	Centromere/kinetochore protein zw10 homolog	ZW10
		0,75	-0,80	0,69	-0,47	Protein DEK	DEK
		1,08	-0,82	0,70	1,29	ATP-binding cassette sub-family F member 2	ABCF2
		0,82	-0,83	0,53	0,68	Ribonucleoprotein PTB-binding 1	RAVER1
		0,78	-0,84	0,93	0,40	Ubiquilin-2	UBQLN2
		0,83	-0,84	0,04	-0,04	MAP7 domain-containing protein 2	MAP7D2
		0,75	-0,89	0,19	-0,23	TAF6-like RNA polymerase II p300/CBP-associated factor-associated factor 65 kDa subunit 6L	TAF6L
	+	0,29	-0,89	2,80	-2,15	Probable ATP-dependent RNA helicase DDX41	DDX41
	+	0,87	-0,90	1,34	-0,66	Interleukin-1 receptor-associated kinase 1	IRAK1
		1,30	-0,91	0,10	0,20	Elongation factor 1-gamma	EEF1G
		1,07	-0,91	0,49	-0,73	Dual specificity tyrosine-phosphorylation-regulated kinase 1A;Dual specificity tyrosine-phosphorylation-regulated kinase 1B	DYRK1A;DYRK1B
		0,22	-0,92	0,72	-0,22	Dolichol-phosphate mannosyltransferase subunit 1	DPM1
		0,64	-0,92	0,40	-1,06	Cyclin-dependent kinase 4	CDK4
		0,44	-0,93	0,72	0,33	Probable ATP-dependent RNA helicase DDX28	DDX28
	+	0,82	-0,95	1,43	2,05	Protein PRRC2B	PRRC2B
		0,21	-0,97	0,05	0,03	78 kDa glucose-regulated protein	HSPA5
		0,35	-0,98	0,14	-0,09	SNW domain-containing protein 1	SNW1
	+	0,25	-0,99	1,37	0,42	60 kDa heat shock protein, mitochondrial	HSPD1
		0,93	-0,99	0,63	-0,58	Melanoma-associated antigen D1	MAGED1
	+	0,59	-0,99	1,51	2,57	Pleckstrin homology domain-containing family A member 5	PLEKHA5

		0,21	-1,00	0,99	0,29	Heterogeneous nuclear ribonucleoprotein K	HNRNPK
		0,91	-1,00	0,99	-1,14	Coiled-coil domain-containing protein 8	CCDC8
		1,18	-1,01	0,12	0,08	Rapamycin-insensitive companion of mTOR	RICTOR
	+	0,82	-1,02	1,34	-0,95	26S proteasome non-ATPase regulatory subunit 3	PSMD3
		0,26	-1,03	0,05	-0,10	Pre-mRNA-processing factor 6	PRPF6
	+	0,32	-1,04	1,54	-1,70	Adenosylhomocysteinase	AHCY
		1,22	-1,06	0,25	0,74	26S protease regulatory subunit 7	PSMC2
		0,90	-1,06	0,54	-0,33	Far upstream element-binding protein 2	KHSRP
		0,64	-1,07	0,93	-1,71	Probable ATP-dependent RNA helicase DDX52	DDX52
		0,27	-1,07	0,14	0,04	Heat shock cognate 71 kDa protein	HSPA8
		0,62	-1,07	0,77	-0,28	Ubiquitin-like modifier-activating enzyme 1	UBA1
		0,47	-1,10	0,01	0,01	Transformation/transcription domain-associated protein	TRRAP
		0,66	-1,11	0,11	0,34	40S ribosomal protein S9	RPS9
	+	0,45	-1,13	1,97	-1,80	T-complex protein 1 subunit theta	CCT8
		0,19	-1,14	0,16	0,32	Actin, cytoplasmic 2;Actin, cytoplasmic 2, N-terminally processed	ACTG1
		0,29	-1,14	1,09	-0,67	Nuclear pore complex protein Nup93	NUP93
		0,27	-1,17	0,86	-0,40	Tubulin alpha chain-like 3	TUBAL3
	+	0,20	-1,17	1,71	-1,07	Poly(U)-binding-splicing factor PUF60	PUF60
		0,66	-1,18	0,02	-0,02	Aurora kinase B	AURKB
		0,61	-1,20	0,52	0,16	Superkiller viralicidic activity 2-like 2	SKIV2L2
	+	1,30	-1,21	2,85	-5,04	RING finger protein 219	RNF219
		0,66	-1,28	0,89	-1,90	60S ribosomal protein L8	RPL8
	+	0,34	-1,32	1,47	0,39	Heat shock 70 kDa protein 1B;Heat shock 70 kDa protein 1A	HSPA1B;HSPA1A
	+	0,87	-1,36	1,56	-0,77	Triosephosphate isomerase	TPI1
		1,03	-1,42	0,56	0,50	Desmocollin-1	DSC1
		0,38	-1,44	0,32	0,37	DNA replication licensing factor MCM3	MCM3
		0,40	-1,45	0,98	-0,56	T-complex protein 1 subunit zeta	CCT6A
	+	0,69	-1,45	1,98	-0,58	Ensconsin	MAP7
		0,51	-1,46	0,05	0,06	Proteasome subunit beta type-2	PSMB2
		0,66	-1,47	0,82	1,43	Apoptosis-stimulating of p53 protein 2	TP53BP2
		0,56	-1,47	0,92	-0,79	Unconventional myosin-Id	MYO1D
	+	0,84	-1,50	3,20	-0,79	Unconventional myosin-Ib	MYO1B
		0,75	-1,55	0,95	-0,51	Dynein assembly factor 5, axonemal	DNAAF5
		0,27	-1,61	0,06	-0,01	Stress-70 protein, mitochondrial	HSPA9
		0,38	-1,64	0,59	0,43	Heterogeneous nuclear ribonucleoprotein M	HNRNPM
		0,85	-1,64	0,83	-0,44	MAP7 domain-containing protein 3	MAP7D3
		0,49	-1,65	0,55	0,35	Heat shock 70 kDa protein 6	HSPA6
	+	1,27	-1,66	1,93	0,95	Centrosomal protein of 131 kDa	CEP131
	+	0,35	-1,68	2,44	1,06	ATP-dependent RNA helicase DDX3X	DDX3X
		0,39	-1,69	0,29	-0,25	Non-POU domain-containing octamer-binding protein	NONO
		0,88	-1,75	0,93	1,63	Zinc finger CCCH-type with G patch domain-containing protein	ZGPAT

	+	0,34	-1,77	1,48	0,83	Probable ATP-dependent RNA helicase DDX5	DDX5
		0,98	-1,81	0,16	-0,17	Protein transport protein Sec16A	SEC16A
		1,19	-1,84	0,29	-0,42	Proteasome subunit beta type-5	PSMB5
		0,52	-1,84	0,03	0,06	Transgelin-2	TAGLN2
		0,53	-1,85	0,31	-0,67	14-3-3 protein zeta/delta	YWHAZ
		0,58	-1,87	1,01	-0,64	Elongation factor 2	EEF2
	+	0,48	-1,88	1,41	0,71	RNA-binding protein 14	RBM14
	+	0,38	-1,93	1,43	0,76	Calcium-binding mitochondrial carrier protein Aralar2	SLC25A13
		0,90	-2,01	0,51	-0,63	Pescadillo homolog	PES1
		0,61	-2,21	1,11	0,32	Splicing factor 1	SF1
		0,54	-2,29	0,94	-0,33	Heat shock protein HSP 90-beta	HSP90AB1
		0,75	-2,33	0,19	-0,32	Ras-related protein Rab-11A;Ras-related protein Rab-11B	RAB11A;RAB11B
		0,98	-2,73	0,32	-0,40	60S ribosomal protein L10a	RPL10A
	+	0,80	-2,89	2,59	-1,16	MAP/microtubule affinity-regulating kinase 3	MARK3
		0,64	-4,05	0,91	0,28	ATPase family AAA domain-containing protein 3A	ATAD3A
		0,69	-4,59	0,47	0,56	Protein FAM46A	FAM46A

Table S1. Mass spectrometry data from FAM46C or D90G immunoprecipitations in the presence or absence of lentiviral envelope and packaging vectors. Related to Fig. 5.

List of the proteins detected in the FAM46C or D90G immunoprecipitations either in the presence (+) or absence (-) of lentiviral envelope and packaging vectors. The fold changes (FCs) are expressed as differences. *P*-values are expressed in logarithmic scale (-log). Significantly differentially abundant proteins are marked with a "+". For each protein detected, the protein name and gene name are listed. Sign: significant; Diff: difference.
